# Site-specific DNA double-strand break induces local transcription *in cis* and protein expression

**DOI:** 10.1038/s42003-026-10230-y

**Published:** 2026-05-19

**Authors:** Alessia di Lillo, Sara Tavella, Fabio Iannelli, Giovanni Crisafulli, Ubaldo Gioia, Lucrezia A. Trastus, Matteo Cabrini, Fabrizio d’Adda di Fagagna

**Affiliations:** 1 IFOM ETS—The AIRC Institute of Molecular Oncology, Milan, Italy; 2 Institute of Molecular Genetics (IGM), National Research Institute (CNR), Pavia, Italy; 3 Present Address: ThermoFisher Scientific—mRNA Department, Monza, Italy; 4 Present Address: Division of Hematopathology, IEO European Institute of Oncology IRCCS, Milan, Italy; 5 Present Address: JoVE, Cambridge, MA USA

**Keywords:** Molecular biology, Cell biology, DNA damage and repair, Translation, Gene expression

## Abstract

The DNA damage response is a complex network of pathways that cells activate to safeguard genome integrity following DNA damage, including DNA double-strand breaks. We and others previously reported that RNA polymerase II, together with components of the preinitiation complex, is recruited to exposed DNA ends. This results in the assembly of a fully competent transcriptional apparatus and the synthesis of damage-induced long non-coding RNAs, which are necessary for full DNA damage response activation. Thus, DNA double-strand breaks could act as transcriptional promoters. Whether such DNA breaks, generated upstream of an open reading frame lacking a transcriptional promoter and followed by a polyadenylation signal, can induce the transcription of a coding RNA that is subsequently translated into a protein product remains unknown. Here, taking advantage of the CRISPR/Cas9 technology, we generate a sequence-specific double-strand break upstream of a promoter-less, and therefore silent, reporter gene in two distinct cellular systems. In both cell models, a DNA double-strand break is sufficient to trigger the expression of polyadenylated transcripts and a protein product. Collectively, our results demonstrate that DNA double-strand breaks can act as functional promoters capable of driving protein synthesis, revealing an additional mechanism through which DNA damage can regulate gene expression.

## Abstract (web summary)

DNA double-strand breaks function as promoter-like elements that drive transcription and protein synthesis of polyadenylated RNAs from otherwise silent DNA regions.

## Introduction

Tens of thousands of DNA lesions have been estimated to occur in a cell every day, constantly threatening the stability of our genome^1^. To safeguard DNA integrity, cells have evolved a complex network of signaling and repair mechanisms, collectively known as the DNA damage response (DDR). DNA double-strand breaks (DSBs), the rupture of both DNA strands, represent one of the most harmful types of DNA lesions^2^. Their sensing activates the DDR apical phosphatidylinositol 3-kinase-like protein kinases (PIKKs) ataxia telangiectasia-mutated (ATM), ataxia telangiectasia and Rad3-related (ATR), and DNA-dependent protein kinase (DNA-PK) that phosphorylate multiple protein targets, including the serine 139 of the histone variant H2AX (γH2AX). This triggers the recruitment of additional DDR proteins such as the p53-binding protein 1 (53BP1) in the form of distinct nuclear foci^2^.

In addition to the well-established role of proteins, several groups including ours demonstrated that RNA plays a role in preserving genome integrity by contributing to DDR signaling and DNA repair in different organisms, including mammals^3–10^. In various experimental systems, we have previously shown that DSBs promote de novo RNA synthesis, acting as functional promoters^11^. Indeed, the catalytic component of RNA Polymerase II (RNAPII) POLR2A is recruited to DNA ends exposed by DSBs together with the main subunits of the transcription preinitiation complex (PIC), the transcription co-activator factor Mediator of RNAPII transcription subunit 1 (MED1) and the elongation factor cyclin dependent kinase 9 (CDK9)^11^. The recruitment of these factors at DSBs is mediated by the DNA ends sensor complex MRE11-RAD50-NBS1 (MRN). These events lead to the synthesis of damage-induced long non-coding RNAs (dilncRNAs) at sites of DNA breaks, effectively establishing a functional transcriptional unit despite the absence of canonical promoter sequences in proximity of the DSB^9–11^. Inactivation of either MRN or PIC components prevents POLR2A recruitment at DSBs and dilncRNA transcription, and this in turn impairs DDR signaling and repair^11,12^. Indeed, dilncRNAs are essential for full DDR activation and DDR foci assembly^12–14^. In particular, RNA drives the recruitment of DDR factors at the site of damage by favoring their liquid-liquid phase separation (LLPS), such as in the case of 53BP1^11^.

Intriguingly, DSB generation and DDR activation have also been reported to promote transcriptional silencing in pre-existing active genes in proximity of the DNA lesion^15–17^. Such phenomenon, also termed DSB-induced silencing in cis (DISC), depends on ATM^15^, DNA-PK^16,18^ and the polycomb repressive complex 1 (PRC1)^19^. We have recently reconciled these apparently contrasting observations by demonstrating that dilncRNA synthesis at DSBs allow the recruitment of the PRC1 component BMI1 to promote H2A-K119 ubiquitination and transcriptional repression. Inhibition of dilncRNAs significantly reduces BMI1 recruitment and DISC formation at damaged genomic loci, demonstrating that DSB generation promotes dilncRNA synthesis that can mediate transcriptional repression of proximal active genes^20^.

Since the generation of a DSB results in the assembly of a functional transcriptional promoter that triggers RNA synthesis, regardless of its genomic location^3,4,21,22^, we tested whether a DSB generated upstream of an otherwise silent transcriptional unit made of an open reading frame (ORF) lacking a transcriptional promoter and followed by a polyadenylation signal (poly(A)) can induce the transcription of a coding RNA that can be translated into a protein product. To address this question, we took advantage of the CRISPR/Cas9 technology as a tool to induce a DSB at specific genomic locations^23–25^.

Here, we show in two experimental cell model systems that a Cas9-induced DSB upstream of a promoter-less reporter gene is sufficient to activate the transcription of a polyadenylated RNA that is subsequently translated in a functional protein. These findings indicate that a DSB alone can promote protein synthesis.

## Results

### Generation and characterization of the HeLa GFP∆Promoter cell system

To test the hypothesis that a DSB can activate the expression of a promoter-less gene unit, we engineered a human cellular system in which a site-specific DSB can be induced upstream of a silent enhanced green fluorescent protein (*EGFP*) ORF followed by a polyadenylation signal but lacking a transcriptional promoter by using the CRISPR/Cas9 technology. This system is composed by the CRISPR-associated endonuclease 9 (Cas9) and a single guide RNA (sgRNA) consisting of a CRISPR RNA (crRNA), containing the sequence complementary to the target DNA, and a trans-activating CRISPR RNA (tracrRNA), that interacts with the Cas9 protein^26^. The genomic target site must be positioned next to a protospacer adjacent motif (PAM, 3-6 bp)^27^. Cas9 is recruited to the target DNA region by the sgRNA and cleaves the DNA upstream the PAM sequence, generating a DSB. The use of a fluorescent reporter gene offers the advantage of monitoring its expression at the single-cell level by fluorescence analyses. We removed the enhancer and promoter regions from the pLenti-CMV-MCS-GFP-SV-puro lentiviral plasmid^28^ by an inverse polymerase chain reaction (PCR) using two divergent primers^25^ which incorporated in their sequence the AGG PAM motif, in this way also introducing a recognition sequence for Cas9 cleavage in the target sequence (Supplementary Fig. 1A). The resulting pLentiGFP∆Promoter lentiviral vector was used to infect HeLa cells. Puromycin selection was exploited to generate a stable cell line, hereafter named HeLa GFP∆Promoter (G∆P) pool cell line, composed by a polyclonal population of cells. Such a cellular system represents a convenient model thanks to random multiple chromosomal integrations, thus reducing the potential contribution from a specific genomic integration site, to a relatively short (720 bp) ORF, and to the possibility to detect events at single-cell resolution by fluorescence imaging. After selection, HeLa G∆P cells were diluted to isolate individual clones of genetically homogeneous cells. In parallel, a cell line was generated by infecting cells with the original vector expressing EGFP under a constitutive strong promoter, from now on named HeLa GFP, as positive control of EGFP expression.

Next, we characterized the number of construct integrations in the pool of HeLa G∆P cells by quantitative PCR (qPCR) by comparing the threshold cycle (Ct) of *EGFP* with that of the β-*ACTIN* gene (*ACTB*)—published copy number profiles of HeLa cells indicate that the genomic region comprising the *ACTB* gene is present in three copies and does not appear to be involved in rearrangements with other portions of the genome that could further increase *ACTB* copy number^29^. Upon lentiviral infection, the number of *EGFP* copies was quantified by qPCR, approximately 18 times higher than the number of copies of the *ACTIN* gene (Supplementary Fig. 1B), although we cannot exclude the possibility of episomal DNA copies. In parallel, we also analyzed HeLa GFP cells that display a similar number of construct integrations as the HeLa G∆P pool (Supplementary Fig. 1B).

Analyses by reverse transcription qPCR (RT-qPCR) for *EGFP* mRNA levels on total RNA from HeLa GFP and from the pool of HeLa G∆P cells confirmed that the absence of the promoter region significantly lowered *EGFP* mRNA levels (Supplementary Fig. 1C). In addition, we monitored EGFP protein expression both at the single-cell level by immunofluorescence (IF) and by western blot (WB) and we consistently observed absence of detectable EGFP expression in the pool of HeLa G∆P cells—HeLa GFP and HeLa wild type (WT) cells were used as positive and negative controls respectively (Supplementary Fig. 1D, E).

Since the *EGFP* RNA transcripts in the pool of HeLa G∆P cells were reduced by ~80% but not completely abolished, likely due also to the contribution of LTR-driven transcription inherent to the lentiviral construct employed^30^, we screened several individual HeLa G∆P clones for number of EGFP construct integrations and *EGFP* mRNA levels. The clone 1 exhibits five times less copies of the construct than HeLa GFP cells (Supplementary Fig. 1F) and, among the clones tested, it is the one with the lowest basal transcription (Supplementary Fig. 1G). Therefore, it was chosen for all further experiments.

Also here, EGFP protein expression was monitored both by IF and by WB and we consistently observed absence of detectable EGFP expression in HeLa G∆P clone 1 and its presence in HeLa GFP cells (Supplementary Fig. 1H,I).

In summary, we generated a suitable cell system to investigate if the generation of a DSB is sufficient to trigger gene expression.

### EGFP detection in HeLa GFP∆Promoter system upon DSB induction

**Fig. 1:**
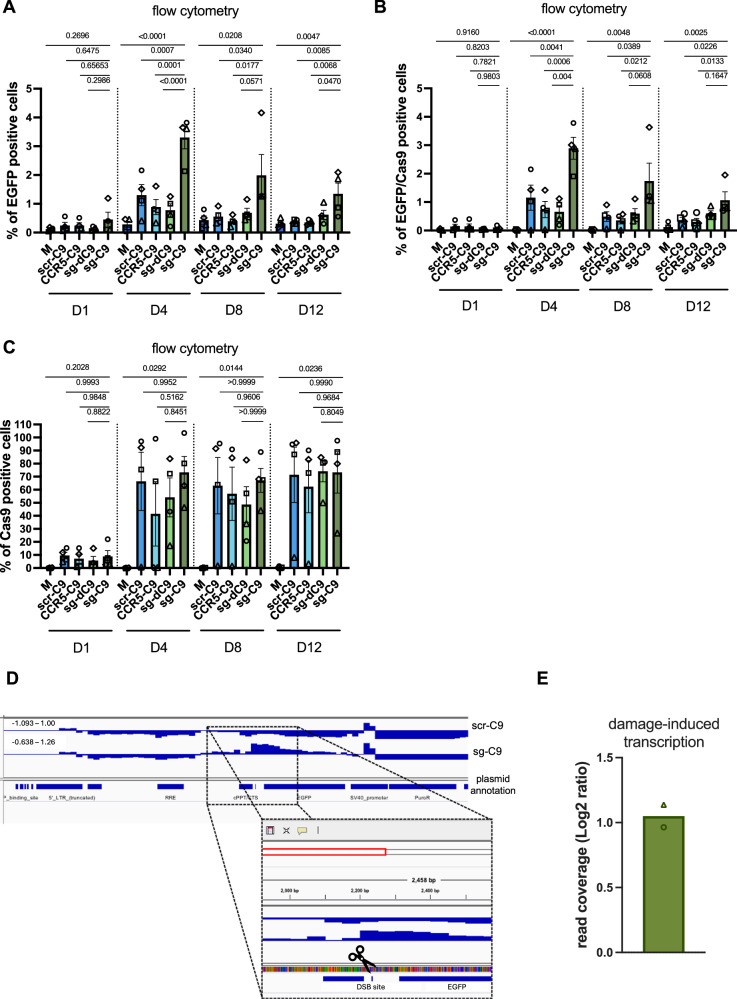
EGFP detection in HeLa G∆P system after lentiviral Cas9 delivery. **A** Quantification of EGFP positive cells by flow cytometry at days 1, 4, 8, and 12 after lentiviral infection. *N* = 4 independent experiments. **B** Quantification of EGFP and Cas9 double-positive cells by flow cytometry at days 1, 4, 8, and 12 after lentiviral infection. *N* = 4 independent experiments. **C** Quantification of Cas9 positive cells by flow cytometry at days 1, 4, 8, and 12 after lentiviral infection. *N* = 4 independent experiments. **D** Graphical representation of the log_2_ ratio of the sg-C9 sample relative to the scr-C9 sample (the clone by a triangle and the pool by a circle). DSB is generated upstream of the EGFP gene by Cas9, represented as scissors. **E** Histogram showing the number of reads in the sg-C9 sample as a ratio normalized on scr-C9, showing an increase of transcripts corresponding to the reads aligning to the region between the DSB site and the *EGFP* coding sequence. *N* = 2 independent experiments, in which the experiment performed in the clone is represented by a triangle, while the experiment performed in the pool is represented by a circle. Data are represented as mean ± SEM.

Having established the experimental setup, we designed a CRISPR/Cas9 sgRNA to match the PAM sequence inserted in the G∆P construct to induce a sequence-specific DSB upstream of the silent *EGFP* ORF and monitor its expression. The sgRNA was cloned in a lentiviral vector engineered to express the Cas9 protein and the sgRNA from the same backbone^31^, from now on referred to as sg-C9. As negative controls, we generated a construct containing a previously validated scramble sgRNA with no predicted match in the human genome (scr-C9)^32^, a construct carrying a sgRNA targeting an unrelated gene (C-C chemokine receptor 5; *CCR5-C9*) whose biallelic inactivation has no detrimental impact on cell survival and proliferation^33,34^, and a construct with the EGFP sgRNA cloned in a vector expressing a catalytically-inactive variant of the Cas9 protein also known as dead Cas9 (sg-dC9)^35^. We therefore infected the selected HeLa G∆P clone 1 with the CRISPR/Cas9 lentiviral vectors expressing scr-C9, CCR5-C9, sg-dC9, and sg-C9. A mock infected condition (M), mimicking the infection process but without the virus, was also included as an additional negative control. To accurately quantify the number and intensity of EGFP-positive cells under the different settings at the single-cell level, we employed flow cytometry analysis (Fig. 1A–C and Supplementary Fig. 1J). Cells were analyzed at different time points up to twelve days post-infection. The analysis performed one day post-infection revealed low numbers of EGFP-positive cells in all the conditions tested, likely too early for viral transduction, robust Cas9 protein expression, DSB induction, and potential EGFP ORF transcription and translation (Fig. 1A–C). Four days after the infection, robust expression of Cas9 was observed (Fig. 1C). Excitingly, up to 3.5% of fluorescent positive cells were observed in HeLa G∆P cells infected with sg-C9, indicating EGFP expression (Fig. 1A, B), while in all the negative control samples EGFP fluorescence remained at background levels, despite similar levels of Cas9 expression (Fig. 1C). EGFP fluorescence induction was consistently observed also at eight and twelve days post-infection exclusively in sg-C9 infected cells, while remaining low in all controls (Fig. 1A, B). This observation was confirmed both in the total cell population and in the population of EGFP/Cas9 double-positive cells, supporting the link between EGFP expression and Cas9 activity (Fig. 1B).

These results obtained by flow cytometry analysis prompted us to investigate events at the RNA level. We therefore extracted and purified the cytoplasmic RNA fraction, enriched in mRNAs, from all conditions and time points tested by flow cytometry, and reverse-transcribed it by using random primers. RT-qPCR for *EGFP* transcripts did not reveal significant differences between the conditions and time points tested (Supplementary Fig 1K). Since HeLa G∆P cells were the result of integrated lentiviral vectors, it is possible that the background transcription observed in all samples was triggered by the known transcription-promoting activity of the lentiviral LTR regions in proximity of the *EGFP* sequence, masking the induction of transcripts induced by DSB^30^.

To circumvent this, we decided to adopt direct cDNA sequencing in the HeLa G∆P clone by Oxford Nanopore Technology (ONT) on a MinION device. This technology generates ultra-long reads that preserve original RNAs length and allows sequencing of full‑length molecules, discriminating between pre‑existing background transcription and newly synthesized RNAs generated after Cas9-induced DNA cleavage. We therefore collected and compared scr-C9 and sg-C9 conditions eight days after infection. To improve the number of relevant reads, the cytoplasmic RNA fraction was first depleted of the ribosomal RNA and processed for direct cDNA sequencing library preparation. The computational analyses of the reads generated confirmed the presence of LTR-driven pre-existing transcripts spanning the full length of the G∆P construct (Supplementary Fig. 1L). To remove such confounding preexisting LTR-driven transcription, we computationally discarded the reads overlapping the DSB site and calculated the log2 ratio between the number of reads present in the sg-C9 condition compared with the those present in the scr-C9 control. In this way, an increase of reads starting in proximity of the break in the sg-C9 sample compared with the negative control became apparent (Fig. 1D). A similar result was observed by analyzing the pool of HeLa G∆P cells treated with either the scr-C9 or the sg-C9. The calculated log_2_ ratio of the read coverage between sg-C9 and scr-C9 samples from the two sets of experiments demonstrated that the DSB generated upstream of the silent *EGFP* gene induces an increase of reads starting precisely from the DSB (Fig. 1E; the log_2_ ratio from the experiment performed in the clone is represented by a triangle, while the log_2_ ratio calculated in the experiment performed in the pool is represented by a circle). The similar behavior observed in the Nanopore sequencing analyses of both HeLa GΔP pool and the isolated clone suggests that the reporter gene activity is not influenced by the integration site of the construct, since the pool contains multiple integrations at different genomic sites, making the result less influenced by a specific genomic context.

Collectively, these results indicate that a DSB induced upstream of an otherwise silent ORF is able to induce the transcription of a RNA that can be translated into a protein, detectable by its fluorescence.

### Generation and characterization of the MEFs Rosa26 EYFP systems

To extend our conclusions and to bypass the intrinsic limitation of the transcriptional noise generated by LTRs in the HeLa G∆P cell system, we decided to test our hypothesis also in immortalized mouse embryonic fibroblasts (MEFs) carrying a single copy of the Lox-Stop-Lox (L-S-L) *EYFP* construct integrated into the *Rosa26* locus, as described^36^. Specifically, Srinivas and colleagues integrated the *EYFP* reporter gene, a 720 bp long sequence very similar to the *EGFP*, immediately followed by a poly(A) signal, in a pBigT plasmid, containing a transcriptional stop sequence flanked by LoxP sites. The authors then integrated a single copy of this plasmid in the murine *Rosa26* locus by homologous recombination (HR). *EYFP* transcription is silenced by a triple Poly(A) (tpA) signal, a strong transcriptional termination sequence, between the promoter and the coding sequence^36^ (Supplementary Fig. 2A). Also in this system, a reporter gene lacking any transcriptional promoter allows to test whether a DSB upstream of it is sufficient to induce its expression, which can be monitored at single-cell level by EYFP fluorescence analysis. Importantly, unlike the HeLa G∆P system, in this case the reporter gene is integrated as a single copy in the genome by HR, minimizing variability due to random lentiviral integration events and copy number differences, without the presence of LTR viral elements that may cause a potentially confounding transcriptional background noise. This allows the integration of the reporter gene in a more controlled and defined genomic context, providing a more reliable interpretation of the reporter behavior.

In this setting, we first tested the lack of transcription of the reporter gene by RT-qPCR, and we observed that the background transcription of the locus was indeed 20 times lower than the previous G∆P system (Supplementary Fig. 2B). We designed a sgRNA to generate a DSB upstream the *EYFP* ORF and we cloned it in a lentiviral vector expressing also the Cas9 protein in the same construct (sg-C9)^31^. Also here, we employed as negative controls a scramble sgRNA (scr-C9)^32^, a sgRNA targeting the mouse CCR5 gene (CCR5-C9)^33,34^ and the EYFP sgRNA cloned in a vector expressing a dead Cas9 (dCas9)^35^.

### EYFP detection in MEFs Rosa26 EYFP system upon DSB induction by lentiviral Cas9 delivery

**Fig. 2:**
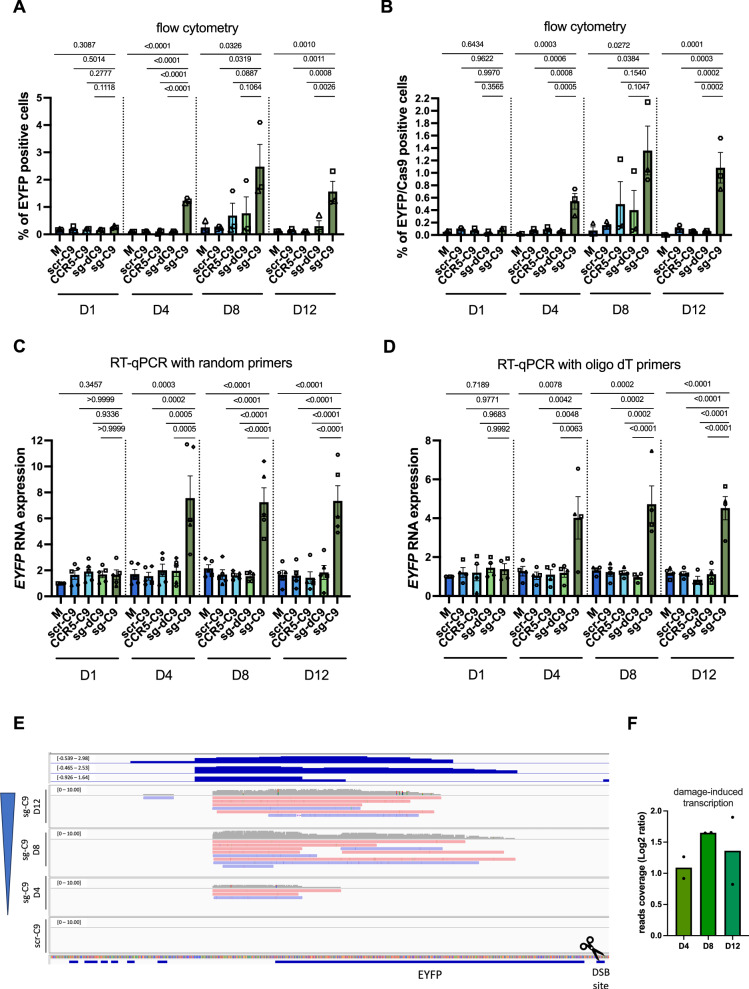
EYFP detection in MEFs R26 EYFP system after lentiviral Cas9 delivery. **A** Quantification of EYFP positive cells by flow cytometry at days 1, 4, 8, and 12 after lentiviral infection. *N* = 3 independent experiments. **B** Quantification of EYFP and Cas9 double positive cells by flow cytometry at days 1, 4, 8, and 12 after lentiviral infection. *N* = 3 independent experiments. **C** RT-qPCR for *EYFP* mRNA expression levels in the cytoplasmic RNA fraction at days 1, 4, 8, and 12 after lentiviral infection. Values were normalized on *B2m* housekeeping gene and shown as relative to mock infected cells at day 1. *N* = 5 independent experiments. **D** RT-qPCR for *EYFP* mRNA expression levels in the cytoplasmic RNA fraction at days 1, 4, 8, and 12 after lentiviral infection. Values were normalized on *B2m* housekeeping gene and shown as relative to mock infected cells at day 1. *N* = 4 independent experiments. **E** Zoomed-in IGV browser visualization of the region of the DSB-induced transcriptional activation at 4, 8, and 12 days after treatment with sg-C9. The histogram in gray shown above the reads represents the coverage. **F** Histogram showing log_2_ ratio of reads in sg-C9 *vs* scr-C9, respectively at 4, 8, and 12 days after DNA damage induction. *N* = 2 independent experiments. Data are represented as mean ± SEM.

After the characterization of this cell system, we sought to test also in this setting whether a DSB upstream of the otherwise silent *EYFP* ORF could induce the transcription of such DNA sequence, leading to protein expression detectable as a fluorescent signal. MEFs were infected with the CRISPR/Cas9 lentiviral vector under the same conditions of the previous system—M, scr-C9, CCR5-C9, sg-dC9 and sg-C9—and analyzed up to twelve days post infection. Excitingly, a EYFP fluorescent signal was detected by flow cytometry exclusively in the sg-C9 sample, starting from four days after Cas9 infection, paralleling a robust induction of Cas9 expression in this sample (Fig. 2A, B and Supplementary Fig. 2C, D). EYFP expression peaked at eight days post-infection with an average of 2.5% of EYFP-positive cells and remained consistently increased up to twelve days upon infection (Fig. 2A, B). In all negative control samples, EYFP fluorescence remained at background levels, despite comparable levels of Cas9 expression (Supplementary Fig. 2C). Notably, only a single copy of the reporter gene is present in this system and it was sufficient to trigger a detectable EYFP signal.

The consistent induction of EYFP protein upon DSB prompted us to investigate the presence of *EYFP* transcripts. Here, the *EYFP* construct was integrated in the genome by HR^36^, thus avoiding viral elements potentially driving transcriptional noise, making this system more suitable for the analysis by RT-qPCR of de novo RNA synthesis from broken DNA ends. To achieve the best detection of the *EYFP* RNA, we collected the cytoplasmic fraction from infected cells at different time points and reverse-transcribed it with random primers. The induction of the *EYFP* RNA was tested by RT-qPCR with primers pairing to the *EYFP* sequence (Fig. 2C). We observed that, while *EYFP* transcription was not yet induced one day after the infection in the sg-C9 sample, consistently with the results obtained by flow cytometry (Fig. 2A, B), from day four onward a robust induction of the *EYFP* RNA was detected and only in the sample infected with sg-C9, compared to all other negative controls. Given the presence of a poly(A) signal downstream of the *EYFP* ORF, we tested whether the *EYFP* RNA molecules detected in the cytoplasmic fraction were also polyadenylated. Therefore, the same RNA preparation was reverse-transcribed using oligo dT primers and RT-qPCR was performed with primers targeting the *EYFP* sequence. Also in this case, starting from day four after the infection, a significant induction of *EYFP* RNA was observed and specifically in the sg-C9 condition only (Fig. 2D). We decided to further characterize these damage-induced transcripts by performing at three different time points (4, 8 and 12 days post infection) a direct cDNA sequencing exploiting Oxford Nanopore Technology (ONT). Since by RT-qPCR *EYFP* RNA induction measured by RT-qPCR was high and consistent starting from four days post-infection till the last timepoint studied, we sequenced all those samples infected with sg-C9 in parallel with GridION 5x in order to reduce variability related to individual library preparation. As a negative control, a scr-C9 sample was also sequenced. Upon DSB generation upstream of the *EYFP* gene, *EYFP* transcripts starting from polyA region were detectable at all the timepoints analyzed, compared with the control scr-C9 condition, that did not show any read at the *EYFP* locus (Fig. 2E, F). Of note, the output of the sequencing revealed that this cell system does not show the background transcription (Supplementary Fig. 2E) we previously observed in HeLa G∆P cells (Supplementary Fig. 1L).

These results confirm that a DSB occurring upstream of a silent ORF leads to the transcription of an mRNA which is polyadenylated—as particularly shown by this last experiment with ONT direct cDNA sequencing—and exported in the cytoplasm, where it is translated into a functional protein.

### EYFP detection in MEFs Rosa26 EYFP system upon RNP-induced DSB

To strengthen our conclusions, we tested a different Cas9 delivery method, based on transfection of a pre-assembled ribonucleoprotein (RNP) complex, consisting of Cas9 protein and sgRNA, thereby avoiding genomic integration of the Cas9 construct. This has emerged as a powerful and popular approach thanks to its advantages of being transient, because of rapid RNP degradation, thus reducing off-target effects, making this strategy the one preferred for in vivo applications^37–39^.

**Fig. 3:**
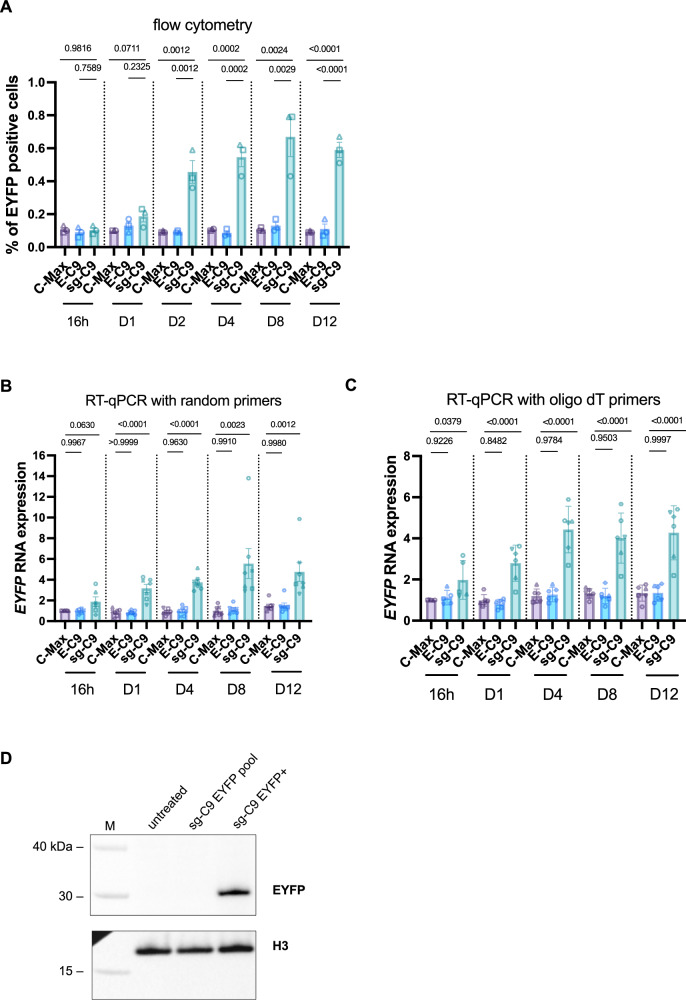
EYFP detection in MEFs R26 EYFP system after RNP delivery. **A** Quantification of EYFP positive cells by flow cytometry 16 h, 1, 2, 4, 8 and 12 days after RNP transfection. *N* = 3 independent experiments. **B** RT-qPCR for *EYFP* mRNA expression levels in the cytoplasmic RNA fraction at 16 h, 1, 2, 4, 8, and 12 days after RNP transfection. Values were normalized on *B2m* housekeeping gene and shown as relative to C-Max treated cells at 16 h. *N* = 6 independent experiments for 16 h timepoint; *N* = 7 independent experiments for all the other time points. **C** RT-qPCR for *EYFP* mRNA expression levels in the cytoplasmic RNA fraction at 16 h, 1, 2, 4, 8, and 12 days after RNP transfection. Values were normalized on *B2m* housekeeping gene and shown as relative to C-Max treated cells at 16 h. *N* = 5 independent experiments for 16 h time point; *N* = 6 independent experiments for all the other time points. **D** Representative immunoblotting of whole cell lysates from untreated, sg-C9 EYFP pool and sg-C9 EYFP+ sorted cells for EYFP protein detection. Cells were collected 12 days after RNP delivery. M = protein marker. Data are represented as mean ± SEM.

Therefore, we tested the impact of a transient DSB induction in the MEFs Rosa26 EYFP system. We compared mock-transfected cells (M), namely those treated only with the transfection reagent, with the empty Cas9-transfected cells (E-C9), which received the Cas9 without the sgRNA, and those transfected with the Cas9 carrying the sgRNA targeting the region upstream the *EYFP* ORF. Cells were analyzed by flow cytometry starting from 16 h post transfection to twelve days afterwards. As expected, the RNP complex was degraded within two days from transfection (Supplementary Fig. 3A, B); however, its transient expression was sufficient to trigger EYFP expression specifically in the sg-C9 condition starting two days after Cas9 transfection, and it remained stably expressed until the last time point studied (Fig. 3A and Supplementary Fig. 3B).

This prompted us to investigate the presence of the *EYFP* transcripts at different time points. Also in this setting, the cytoplasmic RNA fraction was reverse transcribed using either random or oligo dT primers. The induction of the *EYFP* RNA was tested by RT-qPCR with primers targeting the *EYFP* sequence (Fig. 3B, C). *EYFP* transcripts were detectable starting from one day after transfection, consistently with the rapid activity of the pre-assembled RNP complex that bypasses the need for its transcription and translation and remained elevated up to twelve days after Cas9 delivery (Fig. 3B, C).

Finally, we decided to investigate the presence of the EYFP protein in samples treated with sg-C9 by immunoblotting, in order to achieve a more complete characterization of the system. As we observed that only a small fraction of cells treated with the sg-C9 became fluorescent, and this might not be sufficient to detect EYFP protein levels by WB, we enriched for these events by sorting EYFP+ cells. To this end, we collected MEFs R26 EYFP cells twelve days after sg-C9 transfection and sorted them based on EYFP signal. EYFP protein expression was assessed by WB in whole-cell lysates of the unsorted population of sg-C9–treated cells, composed of both EYFP- and EYFP+ cells (sg-C9 EYFP pool), and the sorted sg-C9–treated cells, consisting exclusively of EYFP+ cells (sg-C9 EYFP + ). Untreated MEFs R26 EYFP cells were used as negative control. A clear EYFP band was detected only in the sg-C9 EYFP+ sample, whereas no EYFP signal was observed in either the sg-C9 EYFP pool or the untreated samples (Fig. 3D).

Overall, these results indicate that when a DSB, even when transient, is generated upstream of a silent ORF, it is sufficient to trigger persistent transcription and subsequent protein production.

### Genomic stability of MEFs R26 EYFP locus cells after Cas9-induced DSB

**Fig. 4:**
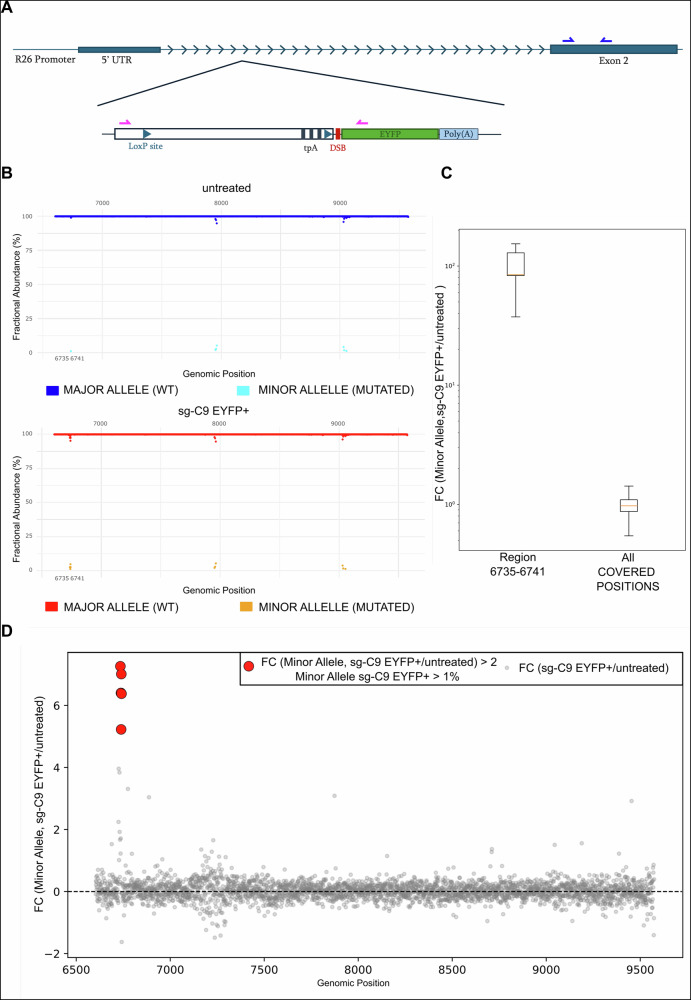
Genomic characterization of the R26 EYFP locus after DSB induction. **A** Schematic representation of primer position on the *R26 EYFP* genomic locus for the PCR experiments performed; the housekeeping gene primers are in blue and the primers spanning the region included into the two LoxP sites are in purple. **B** Fractional abundance of major (wt) and minor (mutated) alleles in both untreated and sg-C9 EYFP+ samples. **C** Comparison of fold changes across the 6735–6741 interval and other covered regions in untreated and sg-C9 EYFP+ samples. Boxplots represent the distribution of log₂ fold-change (log₂FC) values. The center line indicates the median, the box the interquartile range (IQR), and whiskers extend to the minimum and maximum values. **D** Fold change of minor-allele frequencies in sg-C9 EYFP+ relative to untreated. The five positions showing a fold change > 2 and a fractional abundance > 1% are highlighted in red. FC fold change.

It has been reported that Cas9-induced DSBs can result in either small insertions-deletions (indels) or large deletions^40^. To exclude the possibility that EYFP expression might result from genomic rearrangements promoting its transcription, either by removing transcriptional inhibitory elements or by juxtaposing heterologous transcriptional promoter regions into proximity with the otherwise silent *EYFP* ORF, we performed both PCR analyses and high-depth DNA sequencing of the *R26 EYFP* locus. Genomic DNA was extracted from the same MEFs R26 EYFP cells used in Fig. 3D, that is: untreated, sg-C9 EYFP pool, and sg-C9 EYFP+ sorted cells. Untreated MEFs WT lacking the *R26 EYFP* construct were added as an additional negative control. PCR was performed with primers spanning the region included into the two LoxP sites (Fig. 4A, purple primers) and PCR products were analyzed by agarose gel electrophoresis to compare amplicon sizes among conditions (Supplementary Fig. 4A). As expected, MEFs WT showed no amplification of the target region due to the lack of the *R26* locus, demonstrating the specificity of the primers used. The PCR product of the expected size was instead observed in all other conditions, indicating that EYFP expression upon generation of DSBs upstream of the reporter gene was not associated with Cas9-induced structural alterations. Amplification of a housekeeping gene (Fig. 4A, blue primers) was included as positive control in all samples (Supplementary Fig. 4B).

To increase the resolution of our analyses of this genomic locus, we performed high-depth sequencing of the PCR products from untreated and sg-C9 EYFP+ samples, covering the genomic interval from nucleotide 6604–9574 of the *R26 EYFP* locus. Initial inspection confirmed that no significant rearrangements or large-scale genomic alterations were detected across the locus. After excluding big genomic variations, we next assessed the presence of low-frequency genomic changes. High-depth sequencing revealed only three short regions showing minor-allele frequencies (MAF) above 1% and below 6% in both untreated and sg-C9 EYFP+ samples (positions 6735–6741, 7952–7963, and 9030–9054; Fig. 4B). Two of these regions (7952–7963 and 9030–9054) displayed an identical pattern of variants and comparable frequencies across conditions, indicating background subclonal heterogeneity unrelated to Cas9 activity. A modest enrichment of minor alleles was observed only in the 6735–6741 region in sg-C9 EYFP+ cells compared with untreated cells; however, these variants remained of low abundance ( < 6%), insufficient to mechanistically explain the observed EYFP expression (Fig. 4C, D).

Although rare structural events occurring outside the amplified interval cannot be entirely excluded within the current experimental framework, these results demonstrated that the generation of a DSB is sufficient to reactivate gene expression without inducing significant genomic rearrangements.

## Discussion

We have previously demonstrated that the generation of a DSB promotes the assembly of a functional promoter at DSB sites, leading to de novo synthesis of dilncRNAs, regardless of the genomic location^11,12,14^.

Here we tested whether the introduction of a DSB upstream of an otherwise not expressed gene could activate transcription of an RNA that, through the incorporation of canonical mRNA features such as an ORF and a polyadenylation signal, might be translated into a functional protein. We demonstrated in three independent experimental models that a DSB induced by Cas9 upstream of a silent reporter gene can act as a functional promoter, inducing the transcription of a coding RNA that is subsequently translated into a protein, in the absence of genomic rearrangements. Only a small portion of DSB-bearing HeLa GΔP and MEF R26 EYFP cells display EGFP/EYFP expression; this is because the transcriptional activity is not strong, in addition, not all damage-induced transcripts are necessarily extended to the polyadenylation signal or exported and translated. Therefore, not all *EGFP/EYFP* transcripts may recapitulate a fully functional mRNA that can be efficiently translated. In addition, although most cells have been lentivirally transduced, DSB induction may not occur uniformly in all cells.

Our results are consistent with studies linking DNA breaks generated by different stimuli at transcriptional elements, including promoters, with expression of genomic loci^41,42^. For example, it has been reported that transient, site-specific DSBs generated by topoisomerase IIβ at the β-estradiol-responsive pS2 promoter following hormone stimulation, along with DDR protein recruitment, activate gene transcription^43^. Similarly, in neurons, topoisomerase IIβ induces DNA damage in the promoters of early-response genes essential for synaptic plasticity, learning, and memory^44^. An additional link between DSB formation and transcriptional activation has been described in estrogen-sensitive breast cancer cells, where the APOBEC3B enzyme promotes cytidine to uracil conversion at gene regulatory regions, generating DNA damage that triggers the recruitment of DNA repair and chromatin remodeling factors to stimulate gene expression^45^. Another study reported that topoisomerase I induces single-strand breaks in enhancer regions following androgen stimulation in prostate cancer cells, facilitating the recruitment of DDR proteins to relieve torsional stress and regulate transcription^46^. Endonucleases involved in the nucleotide excision repair pathway, such as XPG, have also been shown to induce DSBs and DNA demethylation of promoter regions to optimize RNA synthesis^47^.

Our finding that finely tuned DSBs can activate gene expression unveils intriguing therapeutic implications: sequence-specific DSB generation could be exploited in cancer to reactivate endogenously silenced tumor suppressor genes, or in the treatment of genetic diseases by inducing the expression of genes that compensate for the affected pathways.

Additional implications can be envisioned in cancer therapy. A crucial part in the treatment for localized cancer is radiotherapy (RT), which induces lethal DNA damage in tumor cells^48^. In addition, accumulating evidence indicates that RT also have immunostimulatory effects as it promotes, among other mechanisms, the release of cytokines and chemokines within the tumor microenvironment. Interestingly, RT has been reported to trigger cancer cells to produce neoantigens, highly immunogenic peptides derived from tumor-specific alterations, that can be recognized by T cells. These effects can in turn enhance immune cell infiltration and stimulate anti-tumor immunity^49^. It is possible that RT-induced DSBs, by triggering transcriptional activation and protein synthesis of otherwise not expressed genes, could lead to the generation of novel proteins in cancer cells that may be treated as neoantigens, thereby stimulating tumor-specific immune cell responses. Further work will be necessary to test this hypothesis.

A key question that remains to be elucidated is how transcription is maintained once the initial DNA damage has been repaired. Indeed, we have shown that acute DSB generation by Cas9 in the form of RNP induces seemingly prolonged *EYFP* transcription, that cannot be explained by underlying genomic rearrangements (Figs. 3 and 4). It has been shown that DDR factors such as KAP1^50^ and PARP1^3,51^ orchestrate chromatin decondensation and subsequent accessibility to the damage sites of a variety of factors, including transcription factors and chromatin remodelers^3,51^. Notably, RNA has been proposed to act as a chromatin-associated scaffold that recruits chromatin-modifying complexes to mediate stable changes in gene expression^52^. Our previous works have demonstrated that DSBs drive the assembly of a fully competent transcriptional machinery, including transcriptional co-activators. This process may facilitate the recruitment of chromatin remodeling factors and the deposition of active chromatin marks that persist even after the repair of the break, leaving an epigenetic footprint that marks the locus as transcriptionally active. In this way, a transient DSB could not only initiate local transcription but also establish a long-term memory of a transcriptionally active epigenetic state. This hypothesis could be in line with an exciting recent publication showing that DNA lesions, by altering the three-dimensional chromatin structure, can leave a lasting signature in post-repair genomic regions which persists through multiple cell divisions, priming for heritable gene expression alterations, even after the full repair of the primary DNA breakage^53^.

Altogether, these results indicate that a DSB could act as a regulatory signal capable of activating gene expression, and that this effect may persist in time.

## Methods

### Cell lines generation

HeLa GFP cells were infected with the pLenti-CMV-MCS-GFP-SV-puro plasmid (#73582, Addgene)^28^, while HeLa GΔP pool cells were infected with the same plasmid, previously depleted of the promoter and enhancer regions by inverse PCR using two divergent primers (sequences are listed in Table 1)^25^. Once obtained stable cell lines by keeping cells in culture under puromycin selection (10 ng/ml), HeLa GΔP pool cells were plated at low dilution to allow individual colony growth. These colonies were then expanded to create a stock of clones. Three clones were tested for the LTR-driven background transcription of the *GFP∆Promoter* locus, and clone 1 was selected for the experiments described in the “Results” section for its low background transcription. The number of *EGFP* construct integrations has been calculated as 2^(Ct actin – Ct EGFP)^. SV40 immortalized MEFs Rosa26 Lox-Stop-Lox EYFP were a kind gift from M. McManus, UCSF.

**Table 1:** List of oligonucleotides sequences

Oligo	Sequence
sgRNA scramble FW	CACCGGCGCGAAGCTTAGGGATAAC
sgRNA scramble RV	AAACGTTATCCCTAAGCTTCGCGCC
sgRNA hCCR5 FW	CACCGACAATGTGTCAACTCTTGAC
sgRNA hCCR5 RV	AAACGTCAAGAGTTGACACATTGTC
sgRNA mCCR5 FW	CACCGTCGATGTCATAGCTATAGGT
sgRNA mCCR5 RV	AAACACCTATAGCTATGACATCGAC
sgRNA EGFP FW	CACCGCGATAAGCTTGGGAGTTCCG
sgRNA EGFP FW	AAACCGGAACTCCCAAGCTTATCGC
sgRNA EYFP FW	CACCGATCGATACCGTCGACCTCGA
sgRNA EYFP FW	AAACTCGAGGTCGACGGTATCGATC
EGFP/EYFP FW	GGCACAAGCTGGAGTACAACTACA
EGFP/EYFP RV	TTCTCGTTGGGGTCTTTGCTCA
ACTIN FW	TGTGGGGTCCTGTGGTGT
ACTIN RV	GAAGGGGACAGGCAGTGAG
RPLP0 FW	GTTGCTGGCCAATAAGGTG
RPLP0 RV	GGGCTGGCACAGTGACTT
B2M FW	CTGCAGAGTTAAGCATGCCAGTA
B2M RV	TCACATGTCTCGATCCCAGTAGA
R26 ex2 PCR FW	GCCACATTTGTAAGCTTCATCCAT
R26 ex2 PCR RV	GGTCCTGCTTGAACATTGCC
R26 EYFP PCR FW	CTGCAGGCCGTAGCCGAA
R26 EYFP PCR RV	GAGGACAAACTCTTCGCGGTC
divergent FW	AGGCTGCAGCGTTTAGTGAAC
divergent RV	CTGCAGCCTCGGAACTCCCAAG

All cell lines were grown under standard tissue culture conditions (37°C, 5% CO_2_) in DMEM supplemented with 10% FBS, 2mM L-Glutamine, and 1% Penicillin/Streptomycin.

PCR-based detection of mycoplasma DNA^54^ and biochemical test to determine the presence of mycoplasma enzymes (Lonza LT07-418) were performed in all cell lines to determine their negativity.

### CRISPR/Cas9 delivery

For the lentiviral experiments, cells were infected with either the lentiCRISPRv2-puro lentiviral vector (#98290, Addgene)^31^ or its catalytically inactive form (lentiCRISPR v2-dCas9, #112233 Addgene)^35^; both plasmids carry Cas9 and the sgRNA on the same backbone. Before infection different sgRNAs were individually cloned in the two plasmids, following the Addgene protocol^25^; the sequence for the scramble sgRNA used as a negative control was obtained from Liao et al.^32^, while the sgRNAs targeting *CCR5* and upstream the *EGFP/EYFP* sequences were designed by using different tools to evaluate efficiency scores and possible off-targets (sequences are listed in Table 1). Stbl3 recombination-deficient bacteria (Invitrogen C7373-03) were transformed with the final plasmids, according to the manufacturer’s instructions. Lentiviral particles were produced by transfecting with calcium phosphate HEK293T cells with the vectors together with the plasmids coding for the lentiviral packaging proteins. Before infecting, viral titer was calculated using the Lenti-X GoStix Plus (Takara, Cat#631281).

For the transfection experiments, the ribonucleoprotein (RNP) complex composed by the Cas9 protein (TrueCut Cas9 Protein v2, ThermoFisher) and the sgRNA were transfected into the cells by using the Lipofectamine CRISPRMAX Cas9 Transfection Reagent (ThermoFisher), according to the manufacturer’s instructions.

### Flow cytometry analysis

For EGFP and EYFP analysis, cells were collected and fixed in formaldehyde 2%, then probed with anti-CRISPR/Cas9 antibody conjugated with Alexa Fluor® 647 (mouse monoclonal, clone 7A9–3A3, Cell Signaling Technology, #48796, 1:100) diluted in PBS 1% bovine serum albumin (BSA) for 1 h at room temperature protected from light. After washing, cells were resuspended in 1x PBS for acquisition. Samples were acquired with Attune NxT (ThermoFisher) using a 488 nm laser and 530/30 filter for EGFP/EYFP, 637 nm laser and 670/14 filter for Cas9. Analysis was carried out using FlowJo 10.10.0 (BD Biosciences). After exclusion of doublets and debris, EGFP/EYFP positive cells were gated based on the appropriate negative control for each experimental setting. Specifically, the mock-treated cells were used as negative controls in the experiments where Cas9 was delivered by lentiviral infection, whereas the EC9 condition was used in RNP-based experiments. For each time point, the gating strategy was defined using the corresponding negative control and the same threshold was then applied to all samples within that condition. At least 1×10^4^ cells were analysed for each sample.

### Nucleic acids extraction and standard RT-qPCR

To evaluate the relative number of *EGFP* copies in HeLa GΔP cells, DNA was extracted by using the DNeasy Blood and Tissue Kit (Qiagen, 69506), according to manufacturer’s instructions. SYBR Green-based qPCR experiments (Roche) were carried out on either Roche LightCycler 480 or the LightCycler 96 systems. Each reaction was performed in technical triplicate (sequences are listed in Table 1).

For standard RT-qPCR experiments, total RNA was extracted from cells with the Maxwell RSC simplyRNA Tissue Kit (Promega), according to the manufacturer’s instructions. Residual DNA contamination was removed with DNase I (Qiagen) treatment. cDNA was generated using the SuperScript VILO (Invitrogen) with random primers. Roche SYBR Green-based RT-qPCR experiments were performed on either Roche LightCycler 480 or Roche LightCycler 96 using either *RPLP0* or *B2m* control genes for normalization. Each reaction was performed in technical triplicate (sequences are listed in Table 1).

To study EGFP/EYFP expression reactivation upon DSB induction, cytoplasmic RNA was extracted by following a published RNA fractionation protocol^55^. cDNA was generated using the SuperScript VILO Reverse Transcriptase (Invitrogen) with either random primers or oligo dT primers. Roche SYBR Green-based RT-qPCR experiments were performed on either Roche LightCycler 480 or Roche LightCycler 96 using either *RPLP0* or *B2m* control genes for normalization. Each reaction was performed in technical triplicate (sequences are listed in Table 1).

### Nanopore

The Direct cDNA Sequencing Kit (SQK-DCS109) was used to prepare cDNA for Nanopore sequencing without PCR amplification steps. The cytoplasmic RNA fraction from HeLa GΔP or MEF R26 EYFP cell lines, infected with either scr-C9 or sg-C9, was extracted using Maxwell RSC simplyRNA Tissue Kit (Promega). After quantification by NanoVue Plus (Biochrom), 5 μg of material for each sample was depleted of the ribosomal RNA using the TruSeq Stranded Total RNA Library Prep Gold from Illumina. An input of 100 ng of poly(A) + RNA was used for library preparation. The complementary DNA strand was synthesized using an oligo dT adapter supplied by the kit. Then, adaptor-motor protein complexes were ligated to cDNA ends to allow cDNA to enter the pores for sequencing. Libraries were loaded on R9 flow cells using the Flow Cell Priming Kit (EXP-FLP002) and sequenced individually on MinION 1X device (HeLa GΔP cell lines) or in parallel on the GridION 5x (MEF R26 EYFP cell lines). Only one strand of the duplex is sequenced, producing 1D reads. Base-calling was performed using Guppy (https://nanoporetech.com/) and the resulting reads were aligned to the GFPΔPromoter plasmid using Minimap2^56^. Reads spanning the DSB site, that could arise from pre-existing LTR-driven background transcription, were removed. The log_2_ ratio between the number of reads of the sg-C9 sample and the scr-C9 was calculated with BamCompare from Deeptools suite^57^.

### GFP imaging

Cells were grown on coverslips and fixed with 4% paraformaldehyde. After washing, cells were stained with DAPI and mounted with Mowiol 4–88 (81381, Sigma-Aldrich). Images were acquired using the widefield Olympus Upright microscope and the MetaVue software. Identical acquisition parameters were used for comparative immunofluorescence analyses.

### Protein extraction and immunoblotting

Proteins were obtained by lysing HeLa and MEF cells in 1x Laemmli buffer (2% SDS, 10% glycerol and 60 mM Tris pH 6.8) and immunoblotting experiments were carried out as follows: whole‑cell extracts were boiled at 95 °C and sonicated for 15 s at low intensity using Bioruptor Next Gen (Diagenode) in a water bath at 4 °C prior to separation on 4–12% gradient SDS–PAGE (ThermoFisher). Proteins were transferred to nitrocellulose membranes and probed over-night using the following primary antibodies: anti-GFP (rabbit polyclonal, Abcam ab290, 1:1000), anti-vinculin (mouse monoclonal, Sigma-Aldrich V9131, 1:5000), anti-H3 (mouse monoclonal, Abcam ab10799, 1:1000). Immunoblot signals were acquired using ChemiDoc™ Imaging Systems (Bio-Rad) and the Image Lab™ 6.1 software (Bio-Rad).

### PCR of the R26 locus

The *R26 EYFP* locus was amplified by PCR using 200 ng of genomic DNA, the *R26 EYFP* PCR primers (sequences are listed in Table 1) and the Q5 High-Fidelity DNA Polymerase (M0491, NEB) under the following conditions: 94 °C for 3’; 35 cycles of 94 °C for 30”, 60 °C for 30”, 72 °C for 4’; 72 °C for 10’. The exon 2 of the *R26* locus, used as control, was amplified with the *R26* ex2 PCR primers (sequences are listed in Table 1) under the following conditions: 94 °C for 3’; 25 cycles of 94 °C for 30”, 60 °C for 30”, 72 °C for 3’; 72 °C for 10’. PCR products were loaded on 1% agarose gel and the bands were purified using the Wizard® SV Gel and PCR Clean-Up System (Promega, A9281) according to manufacturer’s instructions.

### Sequencing of the R26 EYFP locus

Fastq files were generated by Illumina Sequencer. Sequencing reads were aligned to the reference genome using BWA-MEM, and per-base allele counts were obtained with the samtools mpileup command according to previous methods^58,59^. For each genomic position of the *R26 EYFP* locus, the number of reads supporting the major (wild-type, WT) and minor (mutated) alleles was extracted for both untreated and sg-C9 EYFP+ samples. The major allele was defined as the most frequently observed nucleotide at that position in the wild-type sample, while the minor allele represented any alternative base differing from the reference.

For each sample, allelic frequencies were calculated as the proportion of reads supporting either the major or minor allele relative to the total coverage at that coordinate. Positions with an allelic frequency below 1% were excluded from percentage-based plots to minimize the contribution of sequencing noise and low-coverage artifacts^60^.

To assess inter-sample variation, the fold change (FC) between sg-C9 EYFP+ and untreated was computed for each position as the ratio of the minor-allele frequencies. Log₂-transformed FC values (log₂FC = log₂[minor_freq₂ / minor_freq₁]) were used to evaluate relative enrichment or depletion of mutated alleles between samples.

Data processing and visualization were performed in R (v4.0.3) using the tidyverse framework (readr, dplyr, tidyr, stringr, ggplot2) and svglite for high-resolution export. Count-based plots were represented as continuous lines, while percentage-based plots were shown as individual points, avoiding artificial interpolation between filtered sites. Both linear and logarithmic Y-axis representations were generated for comparability across a wide dynamic range of allelic frequencies.

### Statistics and reproducibility

All experiments were performed with at least three independent biological replicates (independent experiments) for each group, unless otherwise stated. P values were calculated with GraphPad Prism 10 software. Statistical analyses were performed with one-way ANOVA with Dunnett post hoc test and represented as the mean ± SEM, unless stated otherwise. Effect size and 95% confidence intervals are reported in Supplementary Data 1.

### Reporting summary

Further information on research design is available in the Nature Portfolio Reporting Summary linked to this article.

## Supplementary information

Transparent Peer Review file

Supplementary Information

Description of Additional Supplementary files

Supplementary Data 1

Reporting summary

## Data Availability

Raw Nanopore data on HeLa GΔP cell system are available in the E-MTAB-16605 dataset (ENA database); raw Nanopore data on MEF R26 EYFP cell system are available in the E-MTAB-16775 dataset (ENA database); raw DNA-sequencing data are are available in the E-MTAB-16485 dataset (ENA database). Uncropped and unedited blot and gel images are provided in Supplementary Figs. 5,6. The source data for Figs. 1,2,3 and Supplementary Figs. 1,2,3 are provided in Supplementary Data 1. The effect size and 95% confidence intervals of Figs. 1,2,3 and Supplementary Figs. 2,3 are provided in Supplementary Data 1. All other data are available from the corresponding author on reasonable request.

## References

[1] Jackson, S. P. & Bartek, J. The DNA-damage response in human biology and disease. *Nature* **461**, 1071–1078 (2009). 19847258 10.1038/nature08467 PMC2906700

[2] d’Adda di Fagagna, F. Living on a break: Cellular senescence as a DNA-damage response. *Nat. Rev. Cancer* **8**, 512–522 (2008). 18574463 10.1038/nrc2440

[3] Michelini, F. et al. From ‘cellular’ RNA to ‘smart’ RNA: Multiple roles of RNA in genome stability and beyond. *Chem. Rev.* **118**, 4365–4403 (2018). 29600857 10.1021/acs.chemrev.7b00487 PMC7717669

[4] Modafferi, S., Esposito, F., Tavella, S., Gioia, U. & Francia, S. Traffic light at DSB–transit regulation between gene transcription and DNA repair. *FEBS Lett.* **599**, 177–189 (2025). 39333024 10.1002/1873-3468.15024 PMC11771567

[5] Vítor, A. C. et al. Single-molecule imaging of transcription at damaged chromatin. *Sci. Adv.* **5**, 1–12 (2019). 10.1126/sciadv.aau1249 PMC6326756 30662944

[6] Liu, S. et al. RNA polymerase III is required for the repair of DNA double-strand breaks by homologous recombination. *Cell* **184**, 1314–1329.e10 (2021). 33626331 10.1016/j.cell.2021.01.048

[7] Böttcher, R., Schmidts, I., Nitschko, V., Duric, P. & Förstemann, K. RNA polymerase II is recruited to DNA double-strand breaks for dilncRNA transcription in Drosophila. *RNA Biol.* **19**, 68–77 (2022). 34965182 10.1080/15476286.2021.2014694 PMC8786327

[8] Jalan, M. et al. RNA transcripts serve as a template for double-strand break repair in human cells. *Nat. Commun.* **16**, 4349 (2025). 10.1038/s41467-025-59510-x PMC12065846 40348775

[9] Burger, K., Schlackow, M. & Gullerova, M. Tyrosine kinase c-Abl couples RNA polymerase II transcription to DNA double-strand breaks. *Nucleic Acids Res* **47**, 3467–3484 (2019). 30668775 10.1093/nar/gkz024 PMC6468493

[10] Nojima, T. & Proudfoot, N. J. Mechanisms of lncRNA biogenesis as revealed by nascent transcriptomics. *Nat. Rev. Mol. Cell Biol.* **23**, 389–406 (2022). 35079163 10.1038/s41580-021-00447-6

[11] Pessina, F. et al. Functional transcription promoters at DNA double-strand breaks mediate RNA-driven phase separation of damage-response factors. *Nat. Cell Biol.* **21**, 1286–1299 (2019). 31570834 10.1038/s41556-019-0392-4 PMC6859070

[12] Michelini, F. et al. Damage-induced lncRNAs control the DNA damage response through interaction with DDRNAs at individual double-strand breaks. *Nat. Cell Biol.* **19**, 1400–1411 (2017). 29180822 10.1038/ncb3643 PMC5714282

[13] Francia, S. et al. Site-specific DICER and DROSHA RNA products control the DNA damage response. *Nature* **488**, 231–235 (2012). 22722852 10.1038/nature11179 PMC3442236

[14] Rossiello, F. et al. DNA damage response inhibition at dysfunctional telomeres by modulation of telomeric DNA damage response RNAs. *Nat. Commun*. **8**, 13980 (2017). 10.1038/ncomms13980 PMC5473644 28239143

[15] Shanbhan, N. M., Rafalska-Metcalf, I. U., Balane- Bolivar, C., Janicki, S. M. & Greenberg, R. A. An ATM-dependent transcriptional silencing program is transmitted through chromatin in Cis to DNA double strand breaks. *Cell* **141**, 970–981 (2010). 20550933 10.1016/j.cell.2010.04.038 PMC2920610

[16] Pankotai, T., Bonhomme, C., Chen, D. & Soutoglou, E. DNAPKcs-dependent arrest of RNA polymerase II transcription in the presence of DNA breaks. *Nat. Struct. Mol. Biol.* **19**, 276–282 (2012). 22343725 10.1038/nsmb.2224

[17] Marnef, A., Cohen, S. & Legube, G. Transcription-coupled DNA double-strand break repair: Active genes need special care. *J. Mol. Biol.* **429**, 1277–1288 (2017). 28363678 10.1016/j.jmb.2017.03.024

[18] Iannelli, F. et al. A damaged genome’s transcriptional landscape through multilayered expression profiling around in situ-mapped DNA double-strand breaks. *Nat. Commun.* **8**, 1–7 (2017). 28561034 10.1038/ncomms15656 PMC5499205

[19] Kakarougkas, A. et al. Requirement for PBAF in transcriptional repression and repair at DNA breaks in actively transcribed regions of chromatin. *Mol. Cell* **55**, 723–732 (2014). 25066234 10.1016/j.molcel.2014.06.028 PMC4157577

[20] Esposito, F. et al. DROSHA, DICER and Damage-Induced long ncRNA control BMI1-dependent transcriptional repression at DNA double-strand break. *Cell Rep*. 10.1016/j.celrep.2025.116605 (2025) 10.1016/j.celrep.2025.116605 PMC12727681 41348540

[21] Sepe, S. et al. Telomeric DNA damage response mediates neurotoxicity of Aβ42 oligomers in Alzheimer’s disease. *EMBO J.* **44**, 6078–6111 (2025). 10.1038/s44318-025-00521-1 PMC12583505 40976786

[22] Lu, Y., Storici, F. & Jeon, Y. The multiple layers of RNA response in double-strand break repair. *Exp. Mol. Med.*10.1038/s12276-025-01572-4 (2025). 41258079 10.1038/s12276-025-01572-4 PMC12686478

[23] Doudna, J. A. & Charpentier, E. The new frontier of genome engineering with CRISPR-Cas9. *Science* (1979). **346**, 1258096 (2014). 10.1126/science.1258096 25430774

[24] Hsu, P. D., Lander, E. S. & Zhang, F. Development and applications of CRISPR-Cas9 for genome engineering. *Cell* **157**, 1262–1278 (2014). 24906146 10.1016/j.cell.2014.05.010 PMC4343198

[25] Tavella, S. et al. Weaponizing CRISPR/Cas9 for selective elimination of cells with an aberrant genome. *DNA Repair (Amst)*. **149**, 103840 (2025). 10.1016/j.dnarep.2025.103840 PMC12086175 40319546

[26] Sternberg, S. H. & Doudna, J. A. Expanding the biologist’s toolkit with CRISPR-Cas9. *Mol. Cell* **58**, 568–574 (2015). 26000842 10.1016/j.molcel.2015.02.032

[27] Jinek, M. et al. A programmable dual-RNA-guided DNA endonuclease in adaptive bacterial immunity. *Science (1979)* **337**, 816–821 (2012). 10.1126/science.1225829 PMC6286148 22745249

[28] Witwicka, H. et al. Studies of OC-STAMP in osteoclast fusion: A new knockout mouse model, rescue of cell fusion, and transmembrane topology. *PLoS One* **10**, 1–25 (2015). 10.1371/journal.pone.0128275 PMC4456411 26042409

[29] Adey, A. et al. The haplotype-resolved genome and epigenome of the aneuploid HeLa cancer cell line. *Nature*10.1038/nature12064 (2013) 10.1038/nature12064 PMC3740412 23925245

[30] Jern, P. & Coffin, J. M. Effects of retroviruses on host genome function. *Annu. Rev. Genet.* **42**, 709–732 (2008). 18694346 10.1146/annurev.genet.42.110807.091501

[31] Stringer, B. W. et al. A reference collection of patient-derived cell line and xenograft models of proneural, classical and mesenchymal glioblastoma. *Sci. Rep.* **9**, 1–14 (2019). 30894629 10.1038/s41598-019-41277-z PMC6427001

[32] Liao, H. K. et al. Use of the CRISPR/Cas9 system as an intracellular defense against HIV-1 infection in human cells. *Nat. Commun.* **6**, 1–10 (2015). 10.1038/ncomms7413 25752527

[33] Lombardo, A. et al. Site-specific integration and tailoring of cassette design for sustainable gene transfer. *Nat. Methods* **8**, 861–869 (2011). 21857672 10.1038/nmeth.1674

[34] Sadelain, M., Papapetrou, E. P. & Bushman, F. D. Safe harbours for the integration of new DNA in the human genome. *Nat. Rev. Cancer* **12**, 51–58 (2012). 10.1038/nrc3179 22129804

[35] Babaei, M. et al. CRISPR/Cas9-based editing of a sensitive transcriptional regulatory element to achieve cell type-specific knockdown of the NEMO scaffold protein. *PLoS One* **14**, 1–18 (2019). 10.1371/journal.pone.0222588 PMC6760803 31553754

[36] Srinivas, S. et al. Cre reporter strains produced by targeted insertion of EYFP and ECFP into the ROSA26 locus. *BMC Dev. Biol.* **1**, 1–8 (2001). 11299042 10.1186/1471-213X-1-4 PMC31338

[37] Kim, S., Kim, D., Cho, S. W., Kim, J. & Kim, J.-S. Highly efficient RNA-guided genome editing in human cells via delivery of purified Cas9 ribonucleoproteins. *Genome Res* **24**, 1012–1019 (2014). 24696461 10.1101/gr.171322.113 PMC4032847

[38] Liang, X. et al. Rapid and highly efficient mammalian cell engineering via Cas9 protein transfection. *J. Biotechnol.* **208**, 44–53 (2015). 26003884 10.1016/j.jbiotec.2015.04.024

[39] Vakulskas, C. A. et al. A high-fidelity Cas9 mutant delivered as a ribonucleoprotein complex enables efficient gene editing in human haematopoietic stem and progenitor cells. *Nat. Med.* **24**, 1216–1224 (2018). 30082871 10.1038/s41591-018-0137-0 PMC6107069

[40] Roidos, P. et al. A scalable CRISPR/Cas9-based fluorescent reporter assay to study DNA double-strand break repair choice. *Nat. Commun*. **11**, 4077 (2020). 10.1038/s41467-020-17962-3 PMC7429917 32796846

[41] Vitelli, V. et al. Recent Advancements in DNA Damage-Transcription Crosstalk and High-Resolution Mapping of DNA Breaks. *Annu. Rev. Genomics Hum. Genet.* **18**, 87–113 (2017). 28859573 10.1146/annurev-genom-091416-035314

[42] Pollina, E. A. et al. A NPAS4–NuA4 complex couples synaptic activity to DNA repair. *Nature* **614**, 732–741 (2023). 36792830 10.1038/s41586-023-05711-7 PMC9946837

[43] Ju, B.-G. et al. A Topoisomerase IIβ–Mediated dsDNA Break Required for Regulated Transcription. *Science (1979)*10.1126/science.1127196 (2006). 10.1126/science.1127196 16794079

[44] Madabhushi, R. et al. Activity-induced DNA breaks govern the expression of neuronal early-response genes. *Cell*10.1016/j.cell.2015.05.032 (2015) 10.1016/j.cell.2015.05.032 PMC4886855 26052046

[45] Periyasamy, M. et al. APOBEC3B-mediated cytidine deamination is required for estrogen receptor action in breast cancer. *Cell Rep.* **13**, 108–121 (2015). 26411678 10.1016/j.celrep.2015.08.066 PMC4597099

[46] Puc, J. et al. Ligand-dependent enhancer activation regulated by topoisomerase-I activity. *Cell*10.1016/j.cell.2015.08.044 (2015) 10.1016/j.cell.2014.12.023 PMC4422651 25619691

[47] Le May, N., Fradin, D., Iltis, I., Bougnères, P. & Egly, J. M. XPG and XPF endonucleases trigger chromatin looping and DNA demethylation for accurate expression of activated genes. *Mol. Cell* **47**, 622–632 (2012). 22771116 10.1016/j.molcel.2012.05.050

[48] Weichselbaum, R. R., Liang, H., Deng, L. & Fu, Y. X. Radiotherapy and immunotherapy: A beneficial liaison? *Nat. Rev. Clin. Oncol.* **14**, 365–379 (2017). 28094262 10.1038/nrclinonc.2016.211

[49] Telarovic, I. et al. Delayed tumor-draining lymph node irradiation preserves the efficacy of combined radiotherapy and immune checkpoint blockade in models of metastatic disease. *Nat. Commun.* **15**, 1–23 (2024). 38951172 10.1038/s41467-024-49873-y PMC11217506

[50] Ziv, Y. et al. Chromatin relaxation in response to DNA double-strand breaks is modulated by a novel ATM-and KAP-1 dependent pathway. *Nat. Cell Biol.* **8**, 870–876 (2006). 16862143 10.1038/ncb1446

[51] Izhar, L. et al. Systematic analysis of factors localized to damaged chromatin reveals parp-dependent recruitment of transcription factors. *Cell Rep*. 10.1016/j.celrep.2015.04.053.A (2015) 10.1016/j.celrep.2015.04.053 PMC4464939 26004182

[52] Holoch, D. & Moazed, D. RNA-mediated epigenetic regulation of gene expression. *Daniel. Nat. Rev. Genet* **16**, 71–84 (2015). 10.1038/nrg3863 PMC4376354 25554358

[53] Bantele, S. et al. Repair of DNA double-strand breaks leaves heritable impairment to genome function. *bioRxiv* 2023.08.29.555258 (2023) 10.1126/science.adk6662. 10.1126/science.adk6662 41196998

[54] Uphoff, C. C. & Drexler, H. G. Detection of mycoplasma contamination in cell cultures. *Curr. Protoc. Mol. Biol.* **2014**, 1–14 (2014). 10.1002/0471142727.mb2804s106 24733240

[55] Nojima, T., Gomes, T., Carmo-Fonseca, M. & Proudfoot, N. J. Mammalian NET-seq analysis defines nascent RNA profiles and associated RNA processing genome-wide. *Nat. Protoc.* **11**, 413–428 (2016). 26844429 10.1038/nprot.2016.012 PMC5152764

[56] Li, H. Minimap2: Pairwise alignment for nucleotide sequences. *Bioinformatics* **34**, 3094–3100 (2018). 29750242 10.1093/bioinformatics/bty191 PMC6137996

[57] Ramírez, F. et al. deepTools2: A next generation web server for deep-sequencing data analysis. *Nucleic Acids Res.* **44**, W160–W165 (2016). 27079975 10.1093/nar/gkw257 PMC4987876

[58] Siravegna, G. et al. Radiologic and genomic evolution of individual metastases during HER2 blockade in colorectal cancer. *Cancer Cell* **34**, 148–162.e7 (2018). 29990497 10.1016/j.ccell.2018.06.004

[59] Battuello, P. et al. Mutational signatures of colorectal cancers according to distinct computational workflows. *Brief. Bioinform*. **25**, bbae249 (2024). 10.1093/bib/bbae249 PMC11116831 38783705

[60] Crisafulli, G. et al. Whole exome sequencing analysis of urine trans-renal tumour DNA in metastatic colorectal cancer patients. *ESMO Open* **4**, 1–9 (2019). 10.1136/esmoopen-2019-000572 PMC7001107 32149725

