# Supplementary material for: Site-specific DNA double-strand break induces local transcription in cis and protein expression
Source: Commun Biol. 2026 May 19;9:1000. doi: 10.1038/s42003-026-10230-y (PMC13389459; doi:10.1038/s42003-026-10230-y)
Supplement: Supplementary file 2 — Supplementary Information [file 42003_2026_10230_MOESM2_ESM.pdf]

Supplementary Material for:

Site-specific DNA double-strand break induces local transcription in cis and protein expression

Alessia di Lillo[1,*,#], Sara Tavella[1,2,*], Fabio Iannelli[1,$], Giovanni Crisafulli[1], Ubaldo Gioia[1,2], Lucrezia A. Trastus[1], Matteo Cabrini[1,§] and Fabrizio d'Adda di Fagagna[1,2]✉

1 IFOM ETS - The AIRC Institute of Molecular Oncology, Milan, Italy
2 Institute of Molecular Genetics (IGM), National Research Institute (CNR), Pavia, Italy
* these authors contributed equally
# present address: ThermoFisher Scientific - mRNA Department, Monza, Italy
$ present address: Division of Hematopathology, IEO European Institute of Oncology IRCCS, Milan, Italy
§ present address: JoVE, Cambridge, MA, USA

✉ Correspondence: fabrizio.dadda@ifom.eu

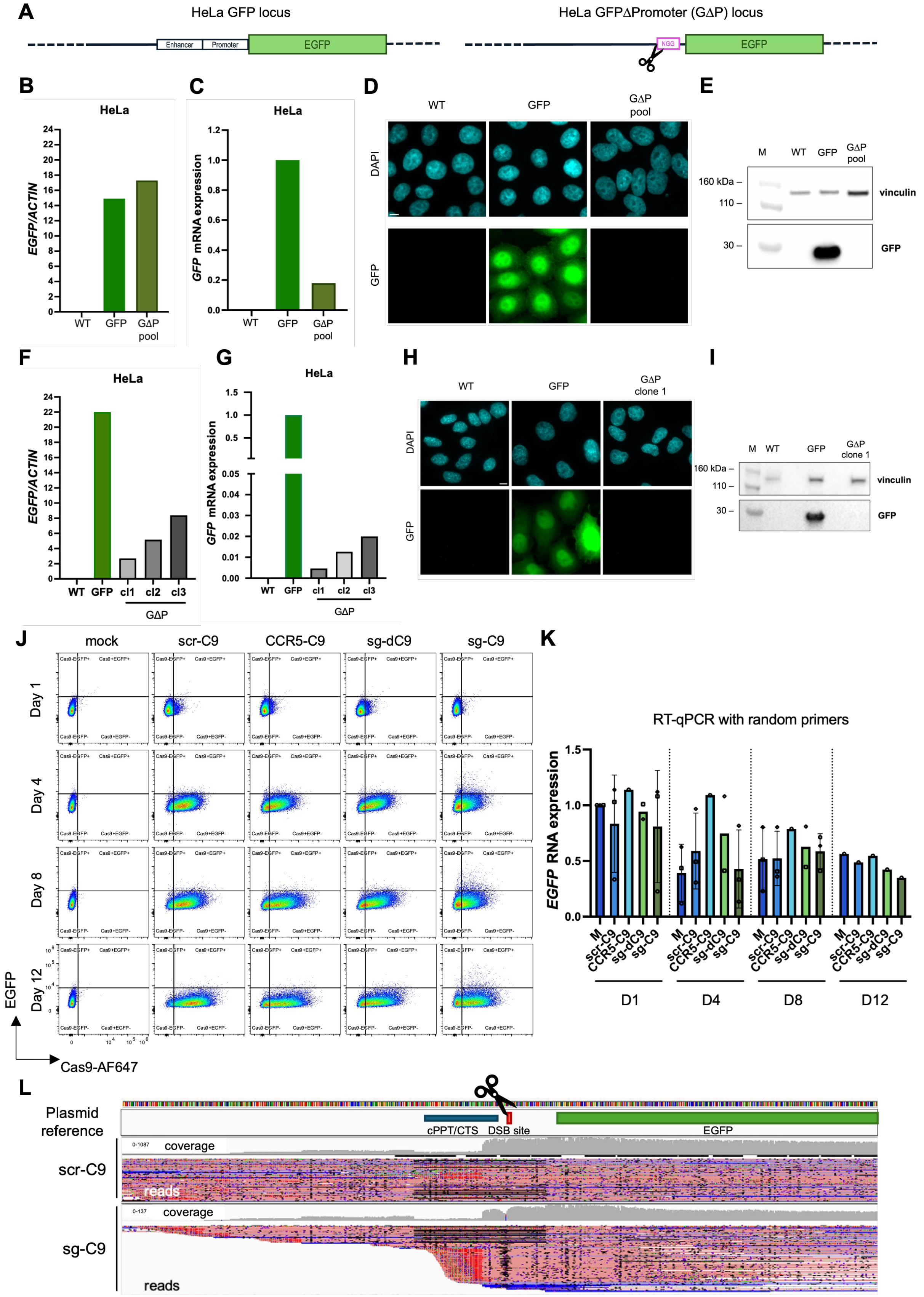

Supplementary figure 1. (A) Schematic representation of the *GFP* and GFPΔ*Promoter* loci. DSBs are generated upstream of the *EGFP* gene by Cas9, here depicted as scissors. **(B)** Relative number of integrated *EGFP* copies in HeLa WT and GΔP pool cells detected by qPCR, compared to the *ACTIN* gene. Mean of N=3 technical replicates. **(C)** RT-qPCR for *GFP* mRNA expression levels in HeLa WT, GFP and GΔP pool cells. Values were normalized on *RPLP0* housekeeping gene and shown as relative to HeLa GFP cells. Mean of N=3 technical replicates. **(D)** Representative immunofluorescence images show lack of EGFP protein in HeLa GΔP pool cells. Scale bar: 10 µm. **(E)** Immunoblotting of whole cell lysates show lack of EGFP protein in HeLa GΔP pool cells. M = protein marker. **(F)** Relative number of integrated *EGFP* copies in HeLa WT and GΔP clones detected by qPCR, compared to *ACTIN*. Mean of N=3 technical replicates. **(G)** RT-qPCR for *GFP* mRNA expression levels in HeLa WT, GFP and GΔP clones. Values were normalized on *RPLP0* housekeeping gene and shown as relative to HeLa GFP cells. Mean of N=3 technical replicates. **(H)** Representative immunofluorescence images show lack of EGFP protein in HeLa GΔP clone 1 cells. Scale bar: 10 µm. **(I)** Immunoblotting of whole cell lysates show lack of EGFP protein in HeLa GΔP clone 1. M = protein marker. **(J)** Representative flow cytometry dot plots showing the gating strategy for EGFP positive (EGFP+Cas9-), Cas9 positive (EGFP-Cas9+) and double positive (EGFP+Cas9+) cells for all conditions at different time points. **(K)** RT-qPCR for *EGFP* RNA expression levels in the cytoplasmic RNA fraction from HeLa GΔP clone at days 1, 4, 8 and 12 after lentiviral infection. Values were normalized on *RPLP0* housekeeping gene and shown as relative to mock infected cells at day 1. N=3 independent experiments for all conditions except for sg-dC9 (N=2), CCR5-C9 (N=1) and day 12 time point (N=1). Data are represented as mean ± SEM. **(L)** IGV browser visualization relative to the GFPΔPromoter plasmid (plasmid reference) of the reads generated by the direct cDNA sequencing of scr-C9 and sg-C9 samples using ONT on a MinION device. The histogram in grey shown above the reads represents the coverage. DSB is generated upstream of the EGFP gene by Cas9, represented as scissors.

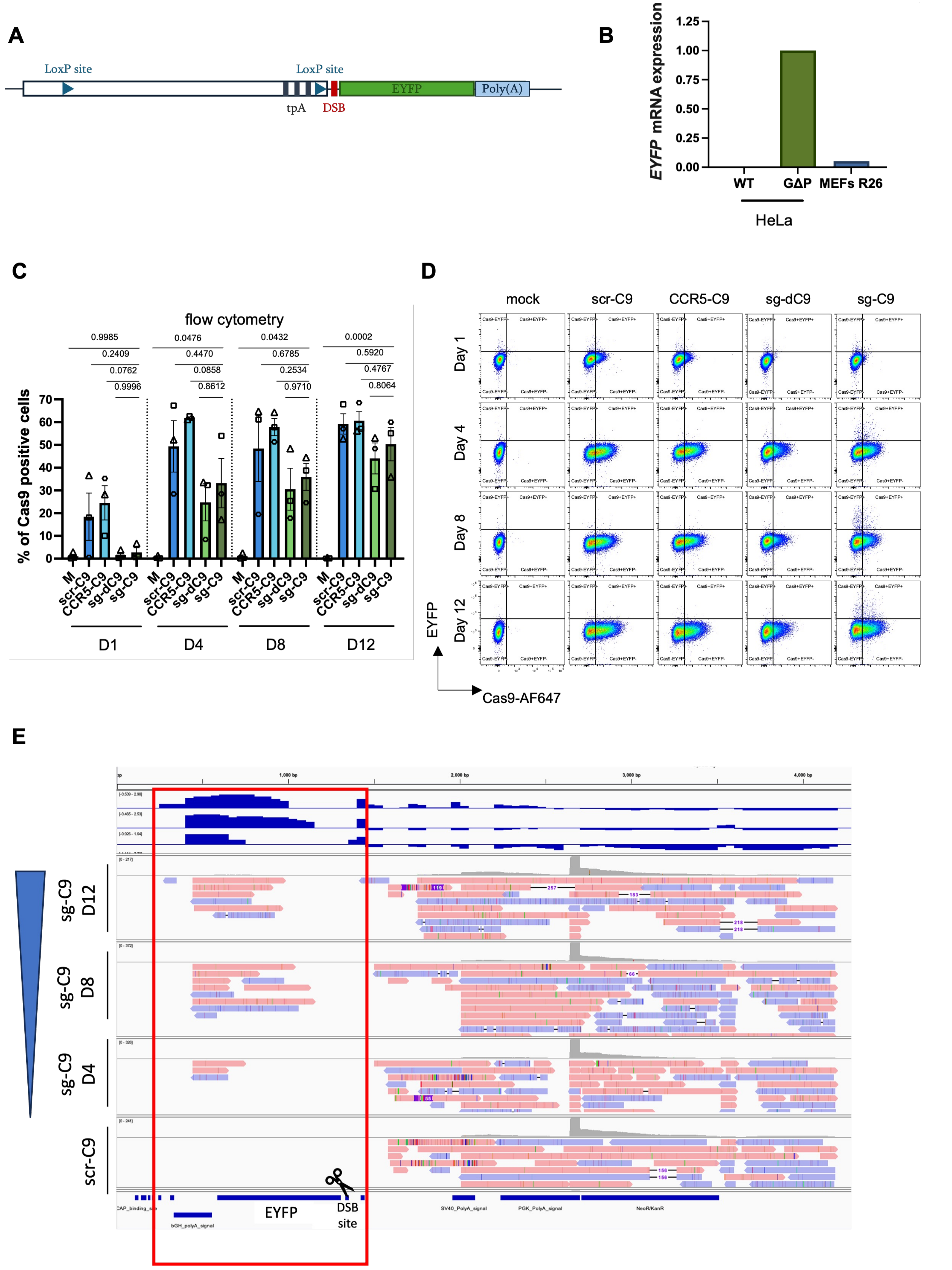

Supplementary figure 2. (A) Schematic representation of the Lox-Stop-Lox (L-S-L) *EYFP* construct, integrated by homologous recombination in the *Rosa26* genomic locus. The site where the DSB was induced by Cas9 is represented in red. **(B)** RT-qPCR to assess the levels of *EYFP* locus background transcription in MEFs R26 EYFP compared to HeLa WT and GΔP cells. Values were normalized on *RPLP0* housekeeping gene and shown as relative to HeLa GΔP cells. Mean of N=3 technical replicates. **(C)** Quantification of Cas9 positive cells by flow cytometry at days 1, 4, 8 and 12 after lentiviral infection. N=3 independent experiments. Data are represented as mean ± SEM. (**D**) Representative flow cytometry dot plots showing the gating strategy for EYFP positive (EYFP+Cas9-), Cas9 positive (EYFP-Cas9+) and double positive (EYFP+Cas9+) cells for all conditions at different time points. **(E)** IGV browser visualization of the reads generated by the direct RNA sequencing of scr-C9 and sg-C9 samples using ONT on a GridION device. The IGV tracks represent reads respectively at 4, 8, and 12 days after DNA damage induction and the scramble as a control. The histogram in grey shown above the reads represents the coverage.

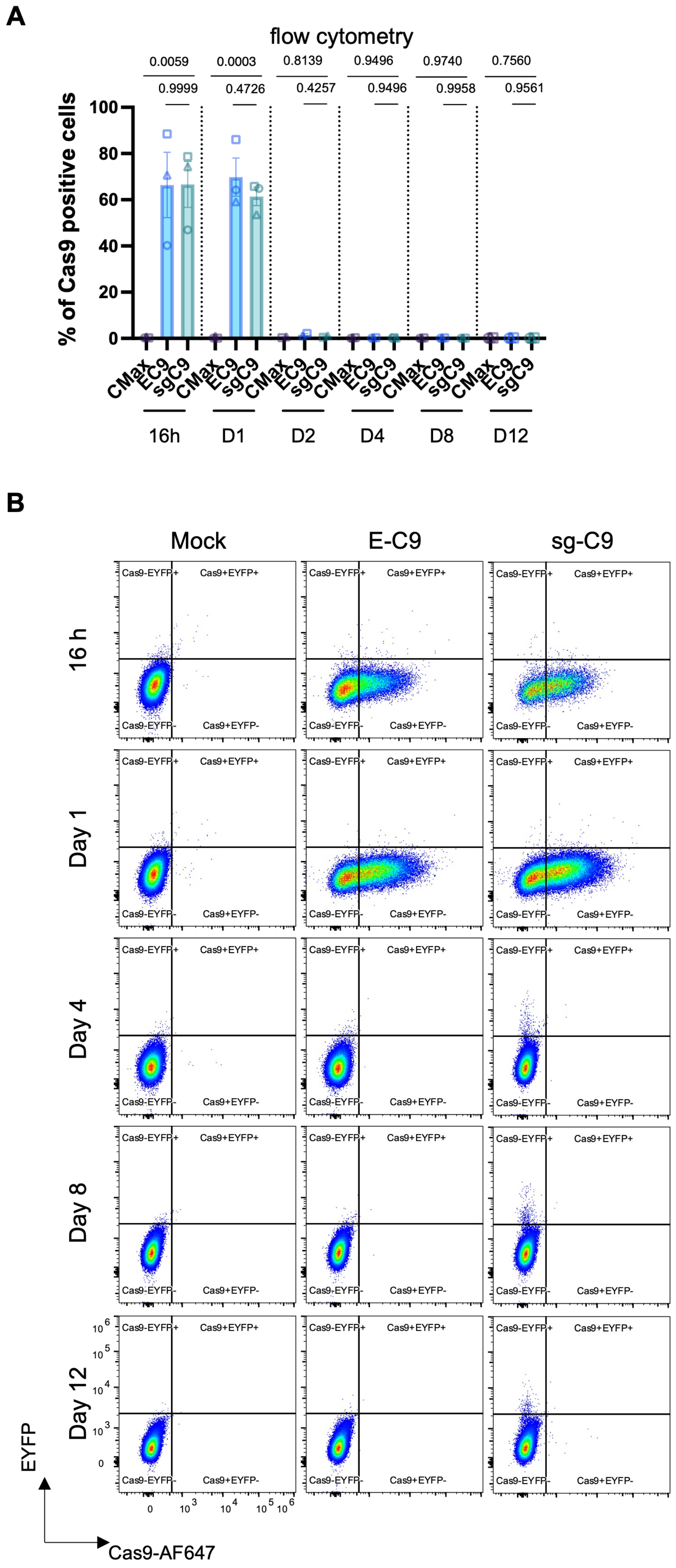

Supplementary figure 3. (A) Quantification of Cas9 positive cells by flow cytometry starting from 16 hours post RNP transfection to twelve days afterwards. N=3 independent experiments. Data are represented as mean ± SEM. **(B)** Representative flow cytometry dot plots showing the gating strategy for EYFP positive (EYFP+Cas9-), Cas9 positive (EYFP-Cas9+) and double positive (EYFP+Cas9+) cells for all conditions at different time points.

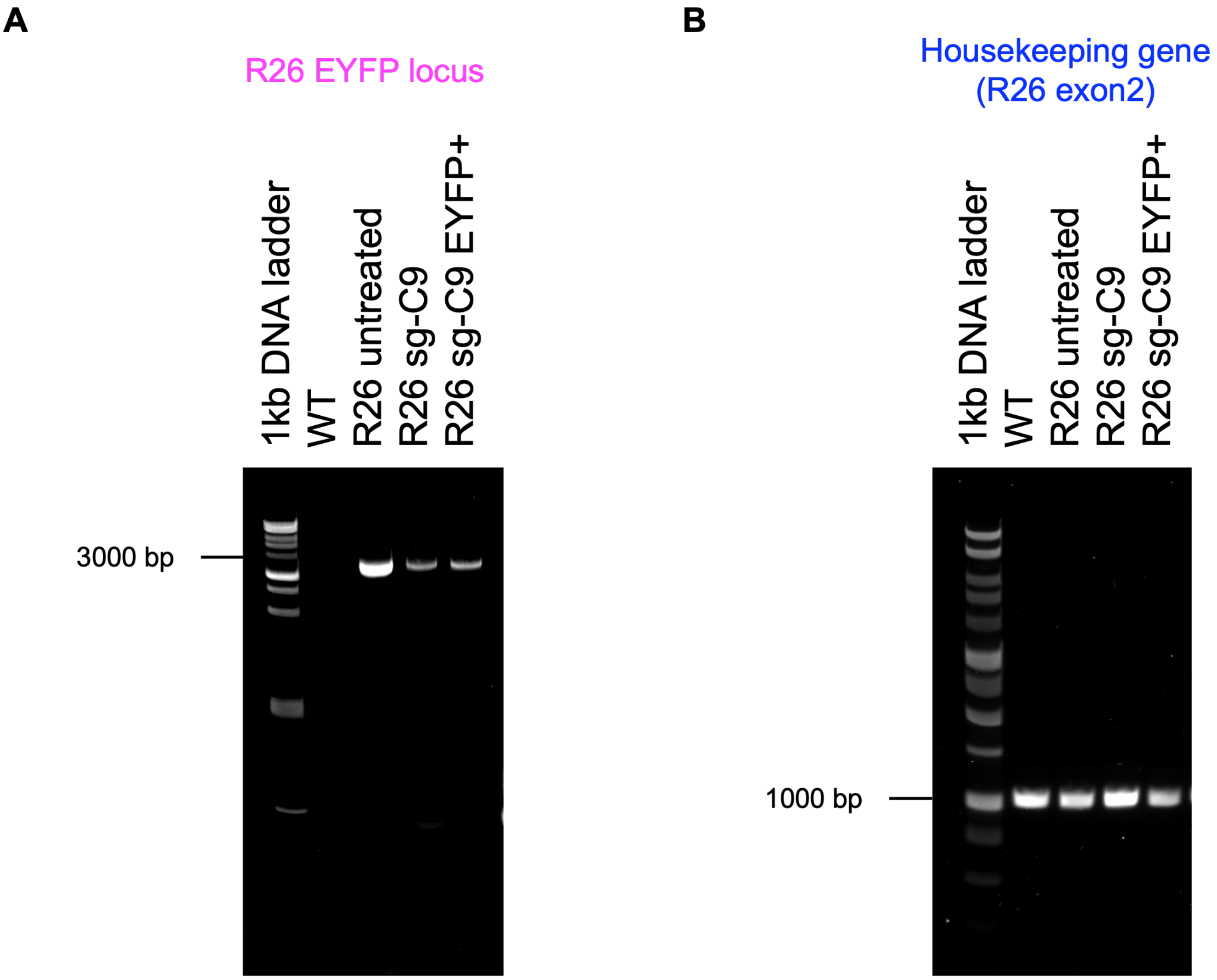

Supplementary figure 4. (A) Agarose gel electrophoresis of PCR products using primers spanning the LoxP sites to verify the preservation of the locus after DSB generation by Cas9. **(B)** Agarose gel electrophoresis of PCR products using primers on the housekeeping gene (the exon 2 of the *Rosa26* locus) to validate the presence of DNA across all samples.

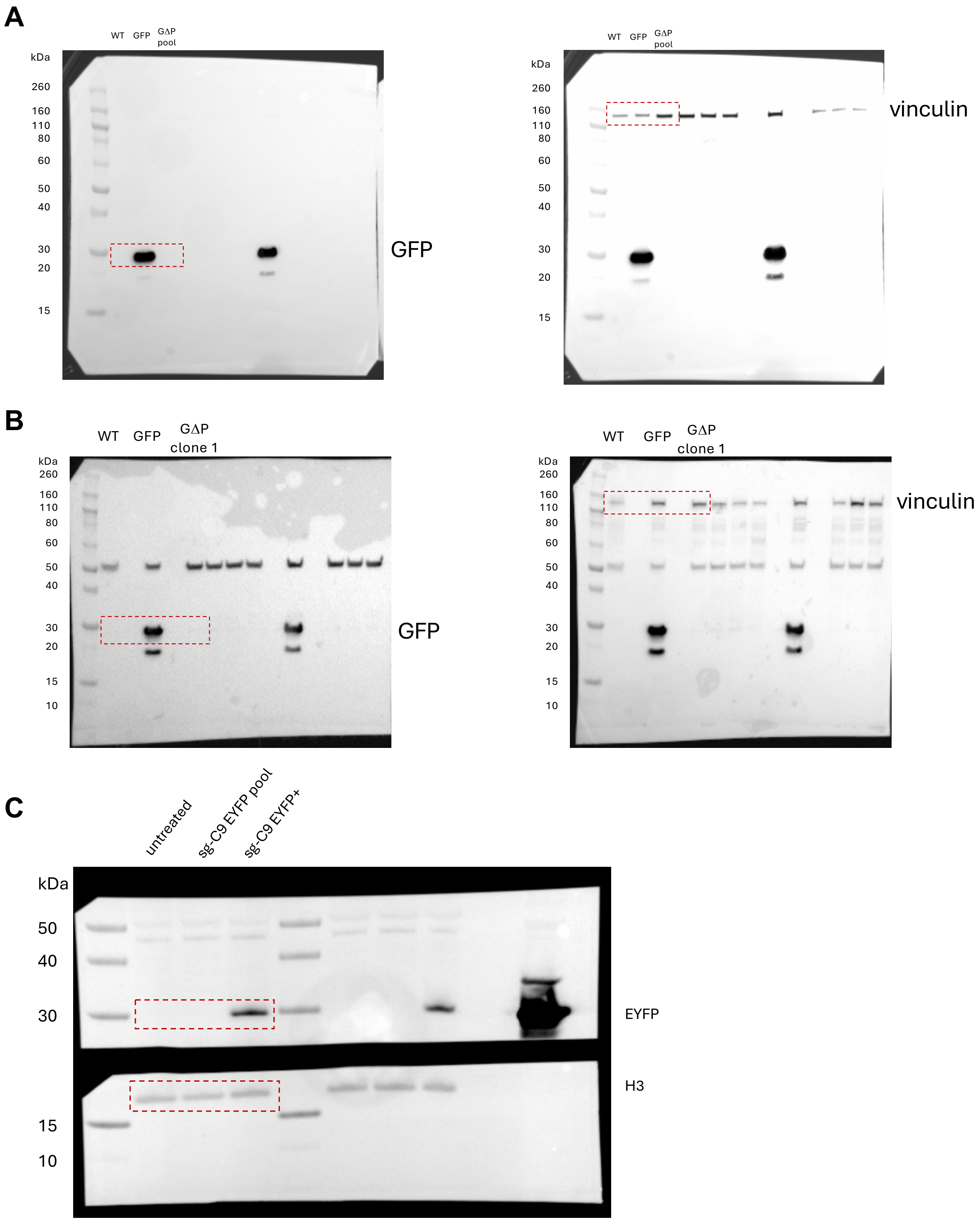

Supplementary figure 5. **(A)** The original, uncropped Western Blot for Fig. S1E. **(B)** The original, uncropped Western Blot for Fig. S1I. **(C)** The original, uncropped Western Blot for Fig. 3D.

A

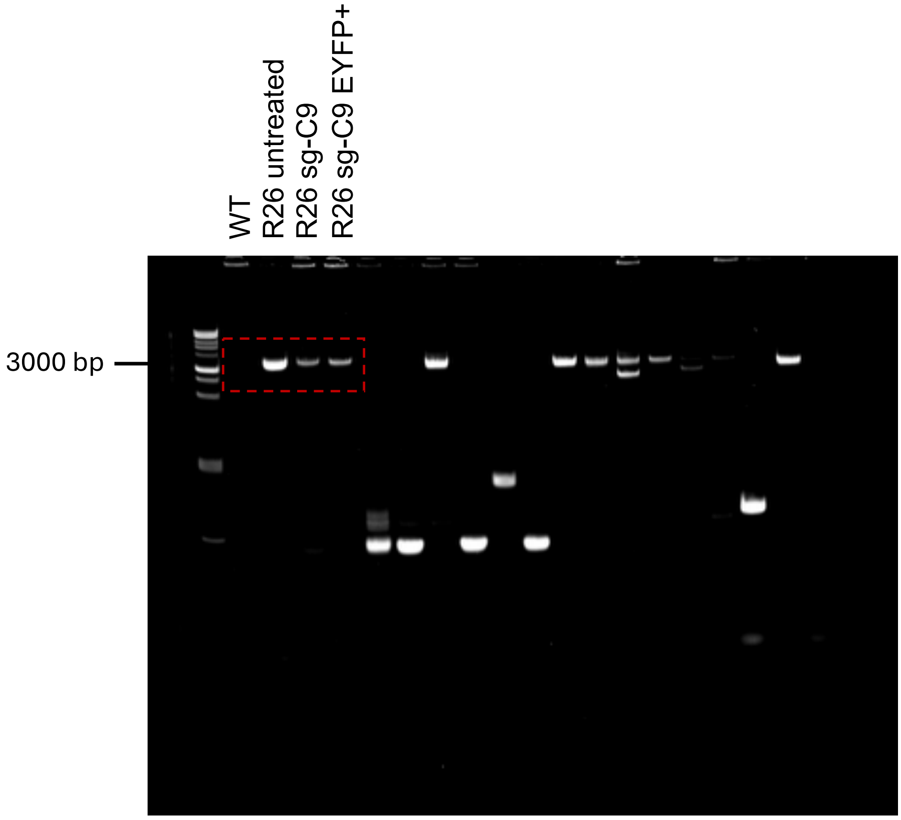

B

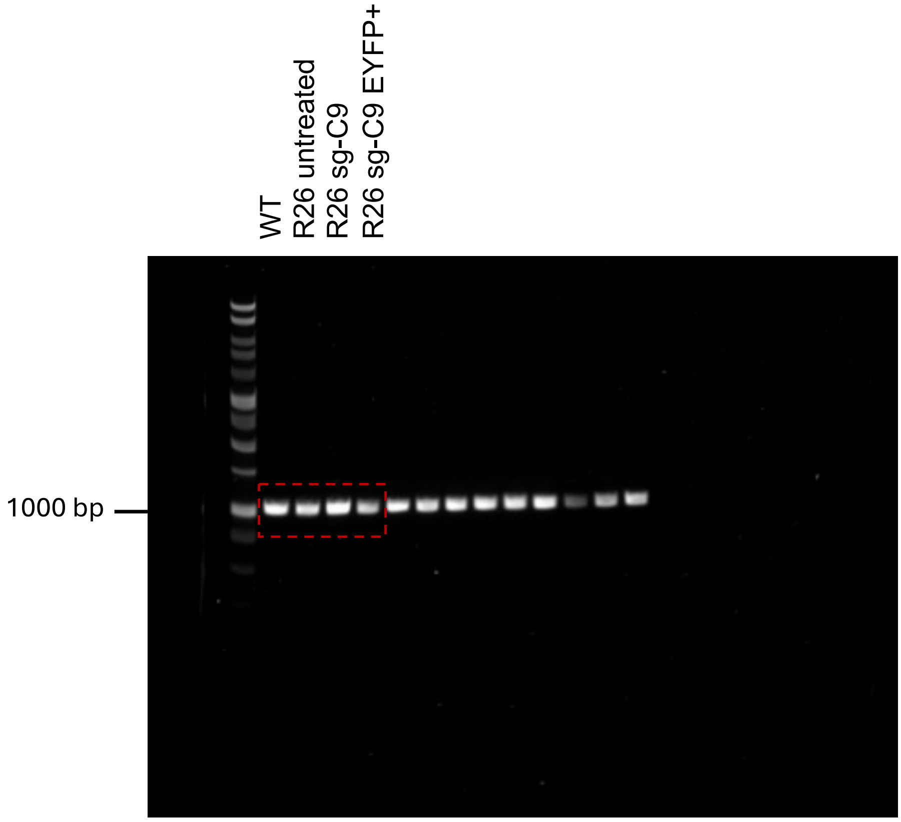

Supplementary figure 6. (A) The original, uncropped Agarose gel for Fig. S4A. **(B)** The original, uncropped Agarose gel for Fig. S4B.
