# Supplementary material for: Site-specific DNA double-strand break induces local transcription in cis and protein expression
Source: Commun Biol. 2026 May 19;9:1000. doi: 10.1038/s42003-026-10230-y (PMC13389459; doi:10.1038/s42003-026-10230-y)
Supplement: Supplementary file 3 — Description of Additional Supplementary files [file 42003_2026_10230_MOESM3_ESM.pdf]

Description of Additional Supplementary Files

File name: Supplementary Data 1

Description: The source data and statistical effect size with 95% confidence intervals behind the graphs in the paper
