# Supplementary material for: Site-specific DNA double-strand break induces local transcription in cis and protein expression
Source: Commun Biol. 2026 May 19;9:1000. doi: 10.1038/s42003-026-10230-y (PMC13389459; doi:10.1038/s42003-026-10230-y)
Supplement: Supplementary file 5 — Reporting summary [file 42003_2026_10230_MOESM5_ESM.pdf]

Corresponding author(s): Fabrizio d'Adda di Fagagna

Last updated by author(s): 21/04/2026

Reporting Summary

Nature Portfolio wishes to improve the reproducibility of the work that we publish. This form provides structure for consistency and transparency in reporting. For further information on Nature Portfolio policies, see our Editorial Policies and the Editorial Policy Checklist.

Statistics

For all statistical analyses, confirm that the following items are present in the figure legend, table legend, main text, or Methods section.

n/a	Confirmed	
☐	☒	The exact sample size (*n*) for each experimental group/condition, given as a discrete number and unit of measurement
☐	☒	A statement on whether measurements were taken from distinct samples or whether the same sample was measured repeatedly
☐	☒	The statistical test(s) used AND whether they are one- or two-sided<br>*Only common tests should be described solely by name; describe more complex techniques in the Methods section.*
☒	☐	A description of all covariates tested
☐	☒	A description of any assumptions or corrections, such as tests of normality and adjustment for multiple comparisons
☐	☒	A full description of the statistical parameters including central tendency (e.g. means) or other basic estimates (e.g. regression coefficient) AND variation (e.g. standard deviation) or associated estimates of uncertainty (e.g. confidence intervals)
☐	☒	For null hypothesis testing, the test statistic (e.g. *F*, *t*, *r*) with confidence intervals, effect sizes, degrees of freedom and *P* value noted<br>*Give P values as exact values whenever suitable.*
☒	☐	For Bayesian analysis, information on the choice of priors and Markov chain Monte Carlo settings
☒	☐	For hierarchical and complex designs, identification of the appropriate level for tests and full reporting of outcomes
☒	☐	Estimates of effect sizes (e.g. Cohen's *d*, Pearson's *r*), indicating how they were calculated

Our web collection on statistics for biologists contains articles on many of the points above.

Software and code

Policy information about availability of computer code

Data collection	Bio-Rad Image Lab 6.1 was used for immunoblot data collection; images at widefield microscope were acquired with MetaVue software; for flow cytometry studies, see the related section. Fastq files were generated by Illumina Sequencer for DNA sequencing and by Guppy for Nanopore experiments.
Data analysis	Bio-Rad Image Lab 6.1 was used for densitometric analysis in immunoblot experiments; GraphPad Prism 10 was used for statistics; for flow cytometry studies, we used FlowJo 10.10.0 (BD Biosciences). For Nanopore analyses, we used Minimap2 and BamCompare (from Deeptools suite). For the analysis of the DNA sequencing, we used BWA-MEM, samtools mpileup and R (v4.0.3).

For manuscripts utilizing custom algorithms or software that are central to the research but not yet described in published literature, software must be made available to editors and reviewers. We strongly encourage code deposition in a community repository (e.g. GitHub). See the Nature Portfolio guidelines for submitting code & software for further information.

Data

Policy information about availability of data

All manuscripts must include a data availability statement. This statement should provide the following information, where applicable:

- Accession codes, unique identifiers, or web links for publicly available datasets
- A description of any restrictions on data availability
- For clinical datasets or third party data, please ensure that the statement adheres to our policy

Raw Nanopore data on HeLa G?P cell system are available in the E-MTAB-16605 dataset; raw Nanopore data on MEF R26 EYFP cell system are available in the E-MTAB-16775 dataset; raw DNA-sequencing data are are available in the E-MTAB-16485 dataset (ENA database). Uncropped and unedited blot and gel images are provided in Supplementary Figs. 5,6. The source data for Figs. 1,2,3 and Supplementary Figs. 1,2,3 are provided in Supplementary Data 1. All other data are available from the corresponding author on reasonable request.

Research involving human participants, their data, or biological material

Policy information about studies with human participants or human data. See also policy information about sex, gender (identity/presentation), and sexual orientation and race, ethnicity and racism.

Reporting on sex and gender	N.A.
Reporting on race, ethnicity, or other socially relevant groupings	N.A.
Population characteristics	N.A.
Recruitment	N.A.
Ethics oversight	N.A.

Note that full information on the approval of the study protocol must also be provided in the manuscript.

Field-specific reporting

Please select the one below that is the best fit for your research. If you are not sure, read the appropriate sections before making your selection.

☒ Life sciences ☐ Behavioural & social sciences ☐ Ecological, evolutionary & environmental sciences

For a reference copy of the document with all sections, see nature.com/documents/nr-reporting-summary-flat.pdf

Life sciences study design

All studies must disclose on these points even when the disclosure is negative.

Sample size	We did not use any criteria to determine the sample size. As much data as possible was collected depending on the nature of the experiments or in order to have statistical analysis.
Data exclusions	Throughout the manuscript no data was excluded.
Replication	For all the experiments at least 3 independent replicates were performed unless differently stated in the figure legends.
Randomization	Wells were randomly assigned into each group and all cells were analyzed equally. Proper controls are present for each experiment.
Blinding	No blinding method was applied, as we used unbiased software for data collection and analysis.

Reporting for specific materials, systems and methods

We require information from authors about some types of materials, experimental systems and methods used in many studies. Here, indicate whether each material, system or method listed is relevant to your study. If you are not sure if a list item applies to your research, read the appropriate section before selecting a response.

Materials & experimental systems

n/a	Involved in the study
☐	☒ Antibodies
☐	☒ Eukaryotic cell lines
☒	☐ Palaeontology and archaeology
☒	☐ Animals and other organisms
☒	☐ Clinical data
☒	☐ Dual use research of concern
☒	☐ Plants

Methods

n/a	Involved in the study
☒	☐ ChIP-seq
☐	☒ Flow cytometry
☒	☐ MRI-based neuroimaging

Antibodies

Antibodies used	anti-Cas9 (7A9-3A3) Mouse Monoclonal Antibody (Alexa Fluor® 647 Conjugate) #48796 Cell Signaling Technology<br>anti-GFP rabbit polyclonal #ab290 Abcam<br>anti-vinculin mouse monoclonal #V9131 Sigma-Aldrich<br>anti-H3 mouse monoclonal #ab10799 Abcam
Validation	All antibodies were validated by the manufacturer and were previously used in peer reviewed works. Methods of validation and references to published application for all antibodies are all present into manufacturer dedicated website page of each indicated product.

Eukaryotic cell lines

Policy information about cell lines and Sex and Gender in Research

Cell line source(s)	HeLa ATCC<br>SV40 immortalized MEFs Rosa26 Lox-Stop-Lox EYFP were a kind gift from M. McManus, UCSF
Authentication	Cell lines were authenticated by STR profiling (GenePrint system, Promega)
Mycoplasma contamination	All cell lines were tested negative for mycoplasma
Commonly misidentified lines (See ICLAC register)	No commonly misidentified lines were used

Plants

Seed stocks	N.A.
Novel plant genotypes	N.A.
Authentication	N.A.

Flow Cytometry

Plots

Confirm that:

☒ The axis labels state the marker and fluorochrome used (e.g. CD4-FITC).

☒ The axis scales are clearly visible. Include numbers along axes only for bottom left plot of group (a 'group' is an analysis of identical markers).

☒ All plots are contour plots with outliers or pseudocolor plots.

☒ A numerical value for number of cells or percentage (with statistics) is provided.

Methodology

Sample preparation	HeLa cells and MEFs were collected and fixed in formaldehyde 2%, then probed with anti-CRISPR/Cas9 AF647-conjugated

Sample preparation	antibody diluted in PBS 1% bovine serum albumin (BSA) for 1 hour at room temperature, protected from light. After washing, cells were resuspended in 1x PBS for acquisition.
Instrument	Attune NxT (ThermoFisher)
Software	FlowJo 10.10.0 (BD Biosciences)
Cell population abundance	At least 10000 cells were analysed for each sample. EGFP/EYFP+ cells varied from around 0% to 3.5%, depending on the experiment and sample. Cas9+ cells varied from around 0% to 75%, depending on the experiment and sample.
Gating strategy	After exclusion of doublets (SSC-H vs SSC-A) and debris (FSC-A vs SSC-A), EGFP/EYFP positive cells were gated based on the appropriate negative control for each experimental setting. Specifically, the mock-treated cells were used as negative controls in the experiments where Cas9 was delivered by lentiviral infection, whereas the EC9 condition was used in RNP-based experiments. For each time point, the gating strategy was defined using the corresponding negative control and the same threshold was then applied to all samples within that condition.

☒ Tick this box to confirm that a figure exemplifying the gating strategy is provided in the Supplementary Information.
