# Supplementary material for: Site-specific DNA double-strand break induces local transcription in cis and protein expression
Source: Commun Biol. 2026 May 19;9:1000. doi: 10.1038/s42003-026-10230-y (PMC13389459; doi:10.1038/s42003-026-10230-y)
Supplement: Supplementary file 1 — Transparent Peer Review file [file 42003_2026_10230_MOESM1_ESM.pdf]

nature portfolio

Peer Review File

Site-specific DNA double-strand break induces local transcription in cis and protein expression

Corresponding Author: Dr Fabrizio d'Adda di Fagagna

This file contains all reviewer reports in order by version, followed by all author rebuttals in order by version.

Version 0:

Reviewer comments:

Reviewer #1

(Remarks to the Author)
In this manuscript, the authors tested whether induction of Cas9-driven DSB is sufficient to activate gene expression and downstream protein synthesis.

While it is established that DNA double-strand breaks can influence RNA polymerase II dynamics, the field contains divergent observations regarding whether DSBs actively recruit Pol II leading to de novo transcription. Numerous studies, including Pessina et al. (Nat Cell Biol. 2019) provide evidence that Pol II and nascent transcripts accumulate at resected DSB ends, supporting a model in which break formation is linked to local transcriptional activity. By contrast, other reports, including Saur et al. (Nat Cell Biol. 2025), challenge this interpretation and instead describe transcriptional repression and RNA:DNA hybrid accumulation at DSB sites, with no clear evidence for productive Pol II recruitment and de novo elongation.

In the context of these conflicting hypotheses, the use of a promoter-less or transcriptionally silenced locus to address the possibility of de novo RNA polymerase II recruitment is, in principle, an intriguing and very important approach, which could provide additional evidence for transcriptional activity around DSBs.

The dataset presented here does not convincingly demonstrate genuine Pol II–dependent transcription initiated at Cs9-driven DBSs in its present form.

Regarding the HeLa GFPΔpromoter cell line, it is not clear how clonal selection was performed, nor whether the resulting HeLa “pool” is monoclonal or polyclonal. This is a major concern given the strong dependence of reporter behaviour on integration site and copy number and this needs to be clarified.

Furthermore, the authors validated the number of EGFP integrations comparing to the Actin gene and assuming to be present in two copies per cell (as stated in ref. 26). This assumption is not valid in HeLa cells. HeLa is highly aneuploid, with extensive copy-number variation across the genome; many loci deviate substantially from diploidy. Therefore, using a “two-copy” reference such as Actin for copy-number estimation in HeLa is intrinsically unreliable unless it is explicitly calibrated against verified diploid reference regions and/or orthogonal copy-number methods. Adey et al. provided a clear reference for the extent of aneuploidy and locus-level copy-number complexity in HeLa (Adey et al., 2013, Nature, DOI: 10.1038/nature12064). This needs to be addressed.

Moreover, qPCR-based copy-number estimation can amplify both episomal and integrated vector sequences, potentially increasing the estimation of number of copies if episomal DNA persists. These limitations are not addressed, yet the copy-number estimate is then used as a foundation for subsequent interpretation.

The HeLa GFPΔpromoter system is not suitable for mechanistic inference because: (i) integration number is uncertain in a highly aneuploid background, (ii) position effects from random lentiviral integration are unavoidable, and (iii) there appears to be limited temporal control of Cas9-induced DSB formation. Any results arising from this HeLa system therefore require substantially more caution than is currently reflected in the manuscript.

If the authors wish to make strong claims about DSB-driven transcriptional activation, they should prioritise the MEF Rosa26

EYFP configuration over the HeLa GFPΔpromoter cell line where integration site and copy number are controlled.

It is unclear why RT–qPCR in Figure 3 was performed on the cytoplasmic fraction rather than total RNA. Given that transcription and polyadenylation occur in the nucleus, limiting the analysis to the cytoplasmic compartment introduces an additional layer of selectivity that is difficult to justify experimentally and may bias the readout towards apparent transcript accumulation. Subcellular fractionation can further introduce variability and cross-contamination that, if not rigorously controlled, risks amplifying marginal signals and favouring an interpretation consistent with the authors' claims rather than providing an unbiased measure of transcriptional output. To mitigate noise and spurious amplification, the protocol should include appropriate controls such as RNase H treatment, no-RT controls, and biological triplicates analysed independently.

Taken together, this an important study, which deserves revision.

Reviewer #2

(Remarks to the Author)
Previous work from the d'Adda di Fagagna lab has shown that DNA double-strand breaks (DSBs) can act as functional promoters by recruiting RNA polymerase II (RNAPII) together with the pre-initiation complex (PIC), Mediator, and the elongation activator P-TEFb (CDK9/cyclin T), leading to de novo RNA synthesis at DNA break sites. This recruitment is mediated by the MRN complex and results in the production of damage-induced long non-coding RNAs that are essential for proper DNA damage response signaling and repair.

This study by di Lillo et al. further expands this concept by showing that Cas9-induced DSBs upstream of a reporter gene lacking a promoter activate gene transcription, leading to functional protein production.

The authors use HeLa cells and MEFs, delivering Cas9 either lentivirally or as pre-assembled ribonucleoprotein complexes. Results are analyzed at the mRNA level (by qPCR) and at the protein level (by Western blot analysis). Finally, the authors show that no genomic rearrangements are observed around the region targeted by Cas9 that could otherwise promote transcription.
Below, I suggest a few minor points that could further improve this already excellent paper prior to publication:

Points

(1) Line 1. The current title is: "Site-specific DNA double-strand break induces protein expression."

This wording is somewhat ambiguous, as DNA damage could also indirectly activate transcriptional programs and induce protein expression, which is not what the authors intend to convey. I suggest considering a revised title such as "Site-specific DNA double-strand break induces gene transcription in cis", or a similar formulation.

(2) Figure 1A, lines 146–147. Can the authors comment on why "only" 3.5% of the HeLa GΔP cells and 2.5% of the MEFs show GFP/YFP expression, assuming that all cells are lentivirally transduced to express Cas9 and sgRNA and therefore should undergo DSB induction? No new experiments are required, but if the authors have examined GFP/YFP expression at the single-cell level, and whether these cells show 53BP1 or γH2AX accumulation at the single Cas9-induced break, this would be nice to include.

(3) It would be beneficial to demonstrate that transcription and GFP/YFP production following Cas9-induced DSBs in one of the systems (perhaps MEFs would be more appropriate) depend on (a) the MRN complex (for example, by pre-treating cells with the MRE11 inhibitor Mirin) and (b) P-TEFb (CDK9/cyclin T), as shown previously in other genomic contexts.

(4) Given that the number of GFP/YFP-positive cells is low, the results are likely sensitive to the gating strategy employed. The description of the gating strategy, including the controls used, should therefore be expanded in the Materials and Methods section.

Reviewer #3

(Remarks to the Author)
This manuscript by di Lillo et al. investigates the impact of a site-specific DNA double-strand break (DSB) on RNA and protein expression. The authors establish two reporter systems: one in HeLa cells, in which a GFP construct lacking a canonical promoter is randomly integrated into the genome together with an upstream viral LTR, and a second in mouse embryonic fibroblasts (MEFs), where a promoterless EYFP reporter is inserted into the Rosa26 locus and transcription is blocked by a Lox-Stop-Lox cassette. In both systems, Cas9-induced DSBs upstream of the reporter lead to transcription and translation of GFP/EYFP in a small fraction of cells.

The authors conclude that DSB induction enables transcription of the GFP/EYFP constructs. These observations are very interesting and consistent with emerging evidence that DNA breaks can stimulate transcription in cis. Importantly, the authors go beyond previous work by proposing that the DSB itself can function as a promoter capable of driving gene expression and protein synthesis. The idea that transcription could initiate directly from the break site, without the need for a

pre-existing promoter to assemble the transcriptional machinery, is conceptually exciting. While I find the conceptual framework compelling and the observations impactful, I believe that the conclusion that DSBs can act as a functional promoter is not yet fully supported by the experimental evidence presented. In particular, the potential contribution of upstream promoter or regulatory elements to GFP/EYFP expression upon break induction has not been sufficiently explored. Below, I outline some experiments that would substantially strengthen the paper.

Major points:

1. As an internal control, it would be important to test whether inducing a Cas9-mediated DSB at an unrelated genomic locus leads to GFP/EYFP expression in the GΔP/EYFP reporter cells. This experiment would help establish the specificity of reporter activation to local break induction.

The main conclusion of the manuscript is that transcription of the GFP/EYFP reporters is initiated directly at the DSB site, independently of upstream promoter activity. However, in both reporter systems, the break is introduced near known regulatory elements (a viral LTR in HeLa cells and the Rosa26 locus in MEFs), raising the concern that DSB induction could indirectly increase transcription from these upstream elements rather than initiating transcription de novo at the break site. While the authors show long-read sequencing of the HeLa construct after sg-Cas9 cutting, similar analyses are not presented for the Rosa26 locus. Several experimental controls could strengthen this claim:

2. The authors should test whether break induction affects transcription upstream of the cut site. RT–qPCR of regions upstream of the DSB (near the LTR in HeLa and the Rosa26 promoter in MEFs) after Cas9 cutting would determine whether basal transcription from these loci increases. Complementary analysis of RNA polymerase II occupancy across the integrated reporter region, including upstream sequences, the cut site, and the reporter gene, by Pol II ChIP would further clarify whether transcription initiates at the break or is enhanced from upstream promoters.

3. The authors use long-read RNA sequencing of the HeLa GΔP construct to distinguish transcripts originating from the upstream LTR versus those initiated at the DSB. However, similar analyses are not presented for the Rosa26 EYFP reporter. Performing comparable long-read RNA sequencing for the Rosa26 locus would allow the authors to determine whether transcription in this system also arises independently of the upstream promoter, thereby strengthening the central claim that break site acts as a functional promoter.

4. In Figure 3, Cas9 is transiently expressed, yet EYFP RNA accumulation is detected several days later, when breaks are likely repaired. This contrasts with previous studies reporting rapid and transient transcriptional responses shortly after DSB induction. To better connect EYFP activation with the DNA damage response, the authors could test whether short-term treatment with Mirin, an MRE11 inhibitor known to suppress DSB-induced transcription, affects EYFP RNA levels.

5. The authors state that high-depth sequencing of sg-Cas9 EYFP$^+$ samples did not reveal significant rearrangements or large-scale genomic alterations, but these data are not shown. Given the low frequency of EYFP-positive cells, it is important to more rigorously exclude the contribution of rare recombination or structural rearrangements at the reporter locus. Providing the sequencing data or complementary analyses (e.g. DNA-FISH with two differently labeled probes flanking the cut site) would strengthen the conclusion that reporter activation reflects local DSB-induced transcription rather than recombination-based events.

Minor points:

6. For clarity, it would be helpful to include the schematic representations of the reporter constructs and the positions of the qPCR primers in the main figures rather than only in the Supplementary Information. This would make it easier for readers to follow the experimental design and data interpretation.

7. The authors report the copy number of the HeLa GΔP reporter (~18 copies per cell), but comparable information is not provided for the WT-GFP construct, which is used side-by-side with GΔP in subsequent experiments. Reporting WT-GFP copy number is important to exclude potential effects of unequal integration on basal or DSB-induced expression.

8. Related to the previous point, for the selected GΔP “clone 1” with low basal transcription, it would be important to indicate the reporter copy number, as this could influence both basal and DSB-induced transcription.

9. The way the authors present the read coverage in Figure S1L is confusing. In Figure S1L, the scale of the coverage is not indicated, and the graph for the scr-Cas9 control appears truncated, making it difficult to compare conditions. In addition, the read coverage for the LTR region is not shown or indicated in the schematic. Including the LTR coverage and marking its position in the scheme, along with a clear y-axis scale for all traces, would improve clarity and help readers better interpret the data.

Version 1:

Reviewer comments:

Reviewer #1

(Remarks to the Author)

The authors have sufficiently addressed all my concerns.

Reviewer #2

(Remarks to the Author)
The authors have addressed all my points and concern adequately.

Reviewer #3

(Remarks to the Author)
The authors have addressed most of my comments satisfactorily, and I have no further concerns.

Open Access This Peer Review File is licensed under a Creative Commons Attribution 4.0 International License, which permits use, sharing, adaptation, distribution and reproduction in any medium or format, as long as you give appropriate credit to the original author(s) and the source, provide a link to the Creative Commons license, and indicate if changes were made.
In cases where reviewers are anonymous, credit should be given to 'Anonymous Referee' and the source.
The images or other third party material in this Peer Review File are included in the article's Creative Commons license, unless indicated otherwise in a credit line to the material. If material is not included in the article's Creative Commons license and your intended use is not permitted by statutory regulation or exceeds the permitted use, you will need to obtain permission directly from the copyright holder.
To view a copy of this license, visit https://creativecommons.org/licenses/by/4.0/

We would like to thank the reviewers and the editor for the constructive comments. Here below, we provide a point-by-point response to the concerns raised. We have included part of these comments in the manuscript.

Reviewers' comments:

Reviewer #1 (Remarks to the Author):

In this manuscript, the authors tested whether induction of Cas9-driven DSB is sufficient to activate gene expression and downstream protein synthesis.

While it is established that DNA double-strand breaks can influence RNA polymerase II dynamics, the field contains divergent observations regarding whether DSBs actively recruit Pol II leading to de novo transcription. Numerous studies, including Pessina et al. (Nat Cell Biol. 2019) provide evidence that Pol II and nascent transcripts accumulate at resected DSB ends, supporting a model in which break formation is linked to local transcriptional activity. By contrast, other reports, including Saur et al. (Nat Cell Biol. 2025), challenge this interpretation and instead describe transcriptional repression and RNA:DNA hybrid accumulation at DSB sites, with no clear evidence for productive Pol II recruitment and de novo elongation.

This referee is correct in pointing to these reports. Indeed, our group too contributed to demonstrate that DSB generation can suppress in cis the expression of pre-existing active genes (Iannelli et al, Nat Comm 2017). For this reason, to provide a rationale for these only apparently contradicting results, we have recently investigated these matters further and reported that damage-induced RNAs control chromatin state and, in this way, dampen the transcription of nearby active genes (Esposito, Capozzo et al, Cell Rep 2025), thus reconciling these observations.

In the context of these conflicting hypotheses, the use of a promoter-less or transcriptionally silenced locus to address the possibility of de novo RNA polymerase II recruitment is, in principle, an intriguing and very important approach, which could provide additional evidence for transcriptional activity around DSBs.

The dataset presented here does not convincingly demonstrate genuine Pol II–dependent transcription initiated at Cs9-driven DBSs in its present form.

Regarding the HeLa GFPΔpromoter cell line, it is not clear how clonal selection was performed, nor whether the resulting HeLa “pool” is monoclonal or polyclonal. This is a major concern given the strong dependence of reporter behaviour on integration site and copy number and this needs to be clarified.
Stable HeLa GΔP pool cells were generated by transducing HeLa WT cells with the pLenti-CMV-MCS-GFP-SV-puro plasmid deleted of the promoter and enhancer regions and by keeping these cells in culture under puromycin selection. As a result, the selected population represents a polyclonal pool of cells. To obtain individual clones, HeLa GΔP pool cells were plated at low density to allow the growth of single, genetically homogeneous, colonies. These colonies were then expanded to create a stock of clones, which were maintained

under puromycin selection. We have revised the manuscript to clarify this aspect; additional details are provided in the methods section.
We agree on the dependence of the reporter behaviour on integration site and copy number; however, the Nanopore sequencing analysis shown in Figure 1D, E was performed on both HeLa GΔP pool and on the isolated clone and showed similar profiles. Furthermore, we also employed the MEF R26 EYFP cells system, in which the reporter gene is integrated as a single copy into the murine Rosa26 locus by homologous recombination. The advantage of this system is to provide a more controlled and defined genomic context, minimizing variability due to random lentiviral integration events and copy number differences, and therefore allowing a more reliable interpretation of the reporter behaviour. The murine Rosa26 locus is a neutral region that has been exploited in very many studies and has become a golden reference and for this reason we have adopted it.

Furthermore, the authors validated the number of EGFP integrations comparing to the Actin gene and assuming to be present in two copies per cell (as stated in ref. 26). This assumption is not valid in HeLa cells. HeLa is highly aneuploid, with extensive copy-number variation across the genome; many loci deviate substantially from diploidy. Therefore, using a “two-copy” reference such as Actin for copy-number estimation in HeLa is intrinsically unreliable unless it is explicitly calibrated against verified diploid reference regions and/or orthogonal copy-number methods. Adey et al. provided a clear reference for the extent of aneuploidy and locus-level copy-number complexity in HeLa (Adey et al., 2013, Nature, DOI: 10.1038/nature12064). This needs to be addressed.
We agree with the concerns of this referee. Unfortunately, it seems we are unable to access the database described in this article (see the screenshot below).

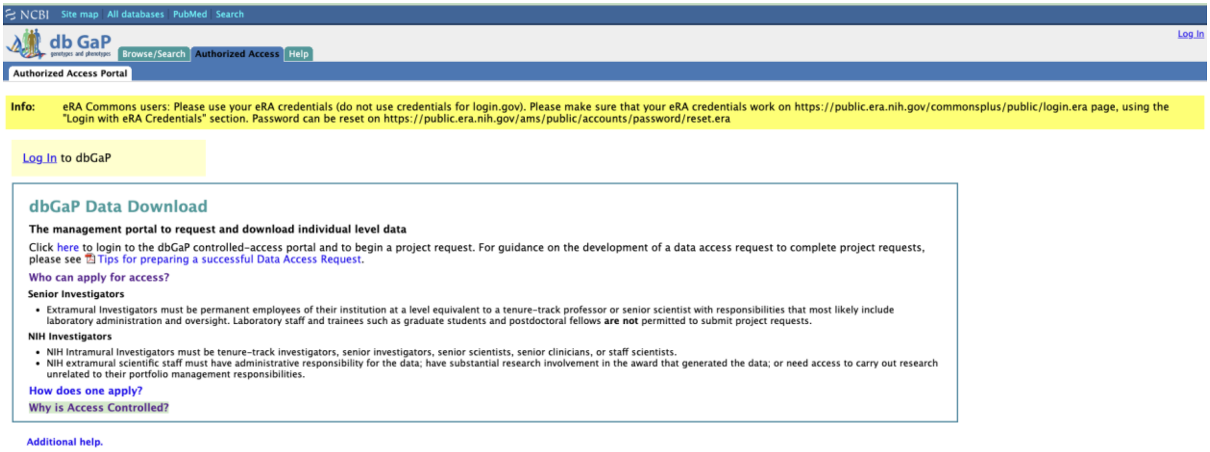

However, in Supplementary Table S7 of this study we found the copy number profile of HeLa cells at kilobase resolution. The β-actin (ACTB) gene is located on chromosome 7p22.1, with genomic coordinates 5,527,148-5,530,601, according to GRCh38 (OMIM/NCBI). In the copy number table, although a small region of 7,619 bp is not covered, the region of interest appears to be present in three copies, suggesting that HeLa cells likely harbor three copies of the ACTB gene. In addition, Figure 1 of the same article indicate that this genomic region does not appear to be involved in rearrangements with other portions of the genome that could further increase ACTB copy number. Given the high degree

of aneuploidy of these cells, we decided not to indicate EGFP copy number but to show the results as the fold change. We have discussed these aspects also in the manuscript.

	A	B	C	D
1	**Table S7 | Copy Number Calls**			
2				
3	Copy number calls at high resolution called using read depth.			
4				
5				
6	**Chr**	**Start**	**End**	**CN**
303	[illegible]	[illegible]	[illegible]	[illegible]
304	chr7	29 843	24 038 252	3
305	chr7	24 038 979	30 044 531	3
306	chr7	30 044 531	30 199 075	2
307	chr7	30 199 075	47 947 924	3
308	chr7	47 947 924	47 964 220	7
309	chr7	47 964 220	51 595 412	3
310	chr7	51 603 031	57 938 791	3
311	chr7	61 058 203	61 078 949	3

However, to have an independent preliminary validation of our conclusions, we have performed fluorescence in situ hybridization (FISH) using a probe against the GFP sequence. Around 16 fluorescent signals per nucleus were counted in HeLa GΔP pool cells, consistent with the copy number estimated by qPCR. Therefore, the number of GFP copies is not far from what we have proposed.

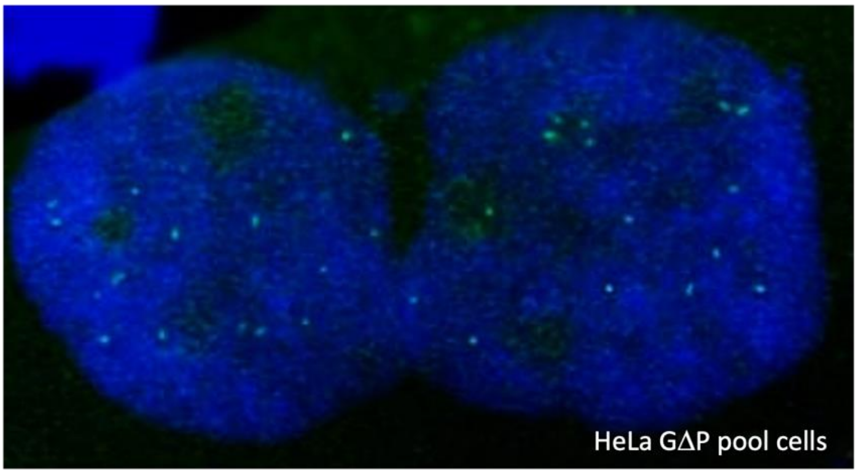

As this is a preliminary experiment, we prefer to keep these data for referee only.

Moreover, qPCR-based copy-number estimation can amplify both episomal and integrated vector sequences, potentially increasing the estimation of number of copies if episomal DNA persists. These limitations are not addressed, yet the copy-number estimate is then used as a foundation for subsequent interpretation.

This is a valid point and we agree with the referee that, based on qPCR-based copy-number estimation, we cannot exclude the possibility of episomal DNA persistence and we have now revised the manuscript accordingly. Nevertheless, the induction of damage-induced transcripts, even if an episomal context, is still informative and supportive of our hypothesis.

The HeLa GFPΔpromoter system is not suitable for mechanistic inference because: (i) integration number is uncertain in a highly aneuploid background, (ii) position effects from random lentiviral integration are unavoidable, and (iii) there appears to be limited temporal control of Cas9-induced DSB formation. Any results arising from this HeLa system therefore require substantially more caution than is currently reflected in the manuscript.
If the authors wish to make strong claims about DSB-driven transcriptional activation, they should prioritise the MEF Rosa26 EYFP configuration over the HeLa GFPΔpromoter cell line where integration site and copy number are controlled.
We agree with the referee that the MEF R26 EYFP cell system is a more reliable and controlled model where more robust conclusions can be drawn and this is the reason why we have used it to support our conclusions.
Regarding the specific concerns on the HeLa GΔP cell system:
(i) we believe that this result is not influenced by the number of integrations of the construct of interest, as it has been confirmed in the MEF R26 EYFP cell system where the construct is present as a single copy integration
(ii) we have performed Nanopore on both HeLa GΔP pool and on the isolated clone and they show similar behaviour (Figure 1D, E). The pool contains multiple integrations at different sites, making the result less dependent on the individual integration sites and therefore less influenced by a specific genomic context. We agree that in the HeLa system the position effect resulting from random lentiviral integrations cannot be eliminated; however, any potential influence of genomic context is internally controlled in our experimental design, since EGFP expression is compared before and after Cas9-induced cleavage
(iii) in this system, Cas9 was delivered by lentiviral infection, avoiding temporal limitations in DSB induction. This is in consistent with the γH2AX data shown in our response to point 2 of referee 2, where persistent DNA damage can be observed for up to 8 days after Cas9 delivery.
We have included these considerations in the manuscript.

It is unclear why RT–qPCR in Figure 3 was performed on the cytoplasmic fraction rather than total RNA. Given that transcription and polyadenylation occur in the nucleus, limiting the analysis to the cytoplasmic compartment introduces an additional layer of selectivity that is difficult to justify experimentally and may bias the readout towards apparent transcript accumulation. Subcellular fractionation can further introduce variability and cross-contamination that, if not rigorously controlled, risks amplifying marginal signals and favouring an interpretation consistent with the authors' claims rather than providing an unbiased measure of transcriptional output. To mitigate noise and spurious amplification, the protocol should include appropriate controls such as RNase H treatment, no-RT controls, and biological triplicates analysed independently.
We have decided to focus our analyses for the detection of damage-induced RNAs on the cytoplasmic fraction to emphasize that these transcripts (previously observed in Michelini et al, NCB 2017 to be predominantly nuclear as shown by different approaches including single-molecule FISH and RT-qPCR on RNAs isolated from the chromatin bound fraction) can be exported to the cytoplasm under specific conditions. This aspect represents the key novel aspect of the present study and the reason why we have intentionally prioritized cytoplasmic analyses.

Regarding the experimental controls required by the referee, the biological replicates shown in this manuscript have been processed and analyzed independently.
To address the additional concerns, we have now controlled for the efficiency of the RNA fractionation protocol by examining the localization of *Malat1*, a nuclear-retained long non-coding RNA. As shown in panel A here below, *Malat1* was predominantly detected in the nuclear fraction, as expected, supporting the correct execution of the RNA fractionation.
In addition, to mitigate noise and spurious amplification, we have also included RNase H treatments, as suggested by the referee. As shown below in panel B, RNaseH treatment did not alter *Eyfp* RNA levels.
Furthermore, we analyzed the nuclear fraction and observed a clear trend toward (although not reaching full statistical significance) the increase of *Eyfp* RNA levels specifically in the sgC9 condition, independent from RNaseH treatment. This is in agreement with the notion that the relatively low abundant *Eyfp* transcripts in the nucleus are rapidly exported into the cytoplasm immediately after their synthesis.
Finally, upon request of referee 2 (see comment 3), we have performed an additional experiment analyzing *Eyfp* transcripts in MEF R26 EYFP cells treated with sgC9 and collected at D1, starting from total RNA. Under these conditions, we were still able to detect their synthesis.
Also, we have included a -RT control. As shown in panel C, the selected wells (in red) display no amplification in the cytoplasmic fraction with the primers used, thereby excluding any DNA contamination.
We believe that these results overall confirm the strengths of our conclusions and, being essentially only controls, we would like to keep them for referees only.

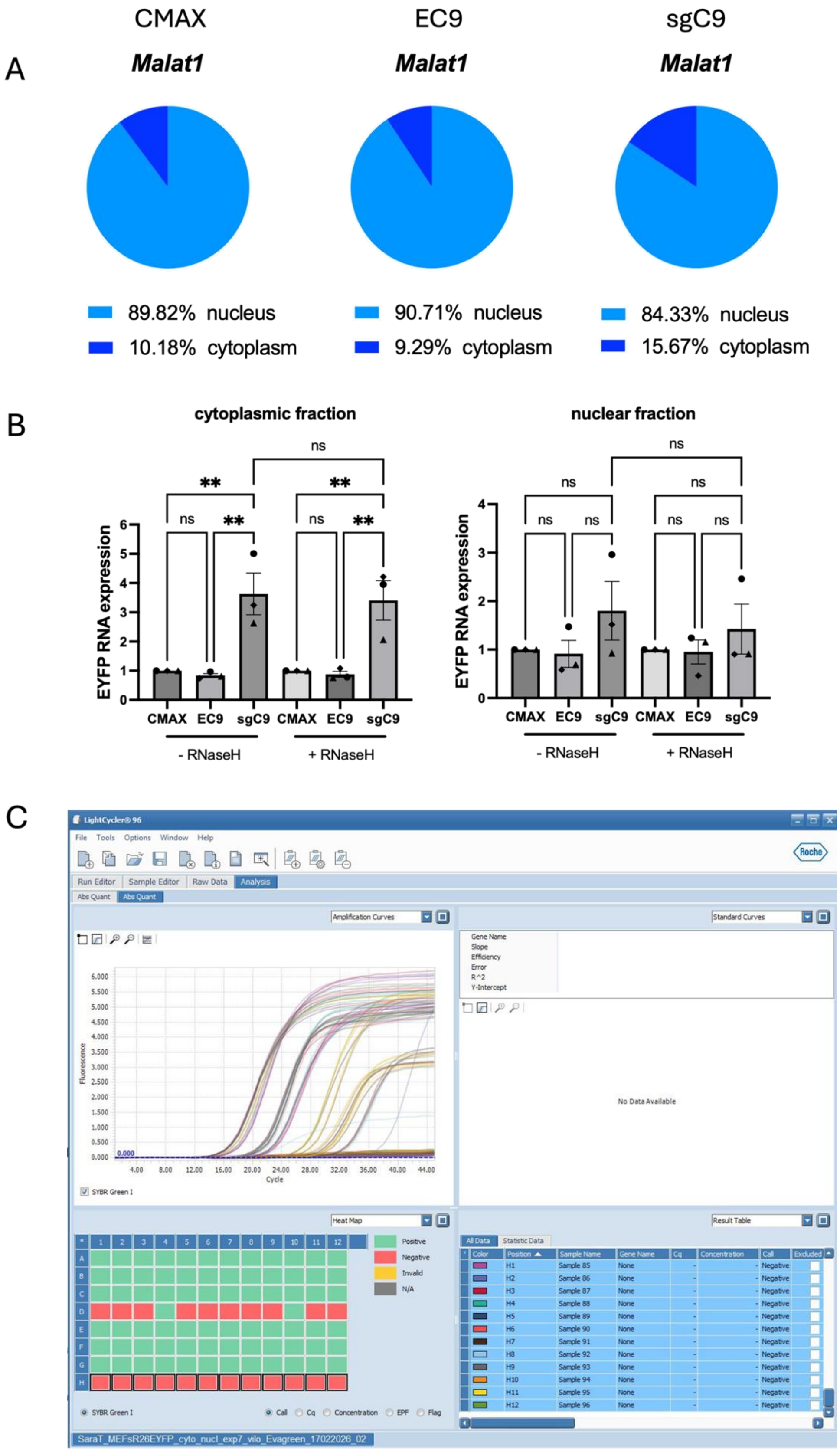

Taken together, this an important study, which deserves revision.

Reviewer #2 (Remarks to the Author):

Previous work from the d'Adda di Fagagna lab has shown that DNA double-strand breaks (DSBs) can act as functional promoters by recruiting RNA polymerase II (RNAPII) together with the pre-initiation complex (PIC), Mediator, and the elongation activator P-TEFb (CDK9/cyclin T), leading to de novo RNA synthesis at DNA break sites. This recruitment is mediated by the MRN complex and results in the production of damage-induced long non-coding RNAs that are essential for proper DNA damage response signaling and repair.

This study by di Lillo et al. further expands this concept by showing that Cas9-induced DSBs upstream of a reporter gene lacking a promoter activate gene transcription, leading to functional protein production.

The authors use HeLa cells and MEFs, delivering Cas9 either lentivirally or as pre-assembled ribonucleoprotein complexes. Results are analyzed at the mRNA level (by qPCR) and at the protein level (by Western blot analysis). Finally, the authors show that no genomic rearrangements are observed around the region targeted by Cas9 that could otherwise promote transcription.
Below, I suggest a few minor points that could further improve this already excellent paper prior to publication:

Points

(1) Line 1. The current title is: "Site-specific DNA double-strand break induces protein expression."

This wording is somewhat ambiguous, as DNA damage could also indirectly activate transcriptional programs and induce protein expression, which is not what the authors intend to convey. I suggest considering a revised title such as "Site-specific DNA double-strand break induces gene transcription in cis", or a similar formulation.
We have now revised the title of the manuscript as follow: "Site-specific DNA double-strand break induces local transcription in cis and protein expression".

(2) Figure 1A, lines 146–147. Can the authors comment on why "only" 3.5% of the HeLa GΔP cells and 2.5% of the MEFs show GFP/YFP expression, assuming that all cells are lentivirally transduced to express Cas9 and sgRNA and therefore should undergo DSB induction? No new experiments are required, but if the authors have examined GFP/YFP expression at the single-cell level, and whether these cells show 53BP1 or γH2AX accumulation at the single Cas9-induced break, this would be nice to include.
We believe that only a small percentage of cells display GFP/YFP expression because not all damage-induced transcripts are likely to be extended to the polyadenylation signal, and not all the polyadenylated RNAs are necessarily exported and translated. Therefore, not all GFP/YFP transcripts may recapitulate a fully functional mRNA that can be efficiently translated. In addition, although the majority of cells have been lentivirally transduced, immunofluorescence analyses revealed a relatively modest increase in γH2AX levels in both cell systems. Indeed, in panel A, sg-C9-treated HeLa GΔP cells show, on average, few more γH2AX foci per cell compared to scr-C9 condition, although this

increase is consistent across all time points analyzed. Also MEF R26 EYFP cells treated with the sg-C9 exhibit a similar increase of γH2AX foci in all time points analyzed, compared to the negative controls (panel B). This suggests that DSB induction does not occur uniformly in all cells, which is consistent with the relatively modest increase in transcription that we have detected. We have also attempted to investigate the colocalization between GFP/YFP and γH2AX by flow cytometry. However, the modest γH2AX signal detected by immunofluorescence was not sufficient to be reliably measured by flow cytometry, making this type of analysis technically unfeasible.

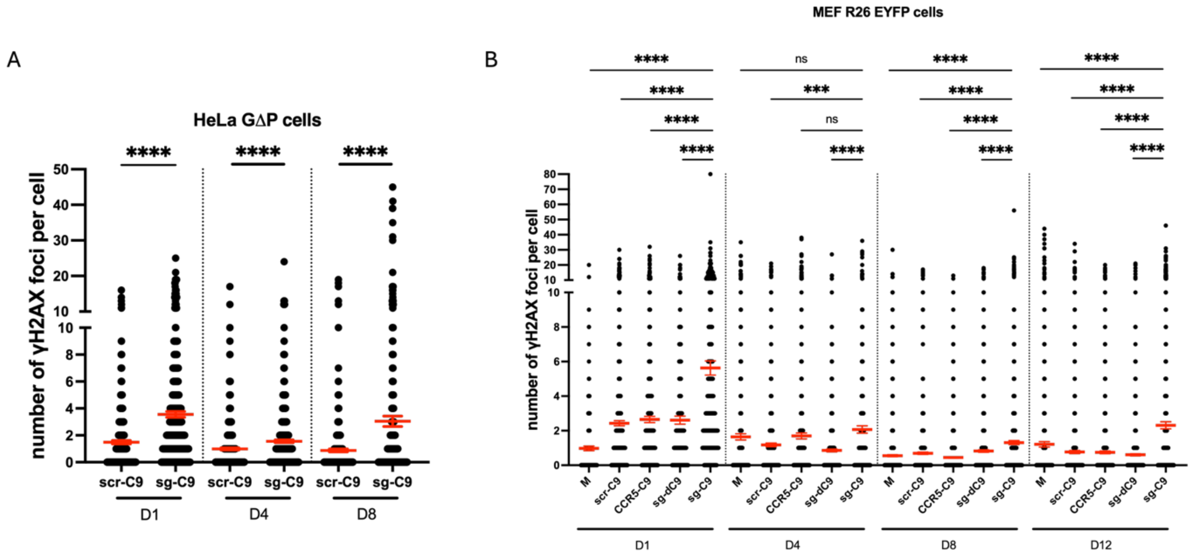

(3) It would be beneficial to demonstrate that transcription and GFP/YFP production following Cas9-induced DSBs in one of the systems (perhaps MEFs would be more appropriate) depend on (a) the MRN complex (for example, by pre-treating cells with the MRE11 inhibitor Mirin) and (b) P-TEFb (CDK9/cyclin T), as shown previously in other genomic contexts.

In previous publications from our laboratory, we have treated cells with mirin (an inhibitor of MRE11) and DRB (5,6-dichloro-1-β-D-ribofuranosylbenzimidazole, an inhibitor of CDK7/9 and consequently of RNA Pol II), demonstrating that damage-induced transcription depends on the MRN complex and is mediated by RNA Pol II (Michelini et al NCB 2017; Pessina et al NCB 2019; Sharma et al Cell Reports 2021). However, in those studies, the experimental setups used involved a temporally controlled and relatively short window of DNA damage generation, compatible with acute mirin and DRB treatments. In contrast, even the fastest experimental design described in this manuscript – based on the RNP-based approach – still requires sustained DNA cleavage for approximately 24 hours before damage-induced transcripts can be robustly detected. Nevertheless, we attempted to follow this referee's suggestion, and in line with our previous experimental settings, we treated cells with mirin (30 minutes) or DRB (2 hours) prior to RNP transfection and collected samples 24 hours later. Under these conditions, cells were therefore exposed to mirin for 24.5 hours and to DRB for 26 hours. Such prolonged treatments resulted to be toxic. We managed to analyze the material from the DRB experiment which supported the evidence for the involvement of RNA Pol II in

the transcription of damage-induced transcripts (see graph below, summarizing two independent biological replicates). However, given the toxicity associated with extended transcriptional inhibition, we prefer to keep these data for referee only.

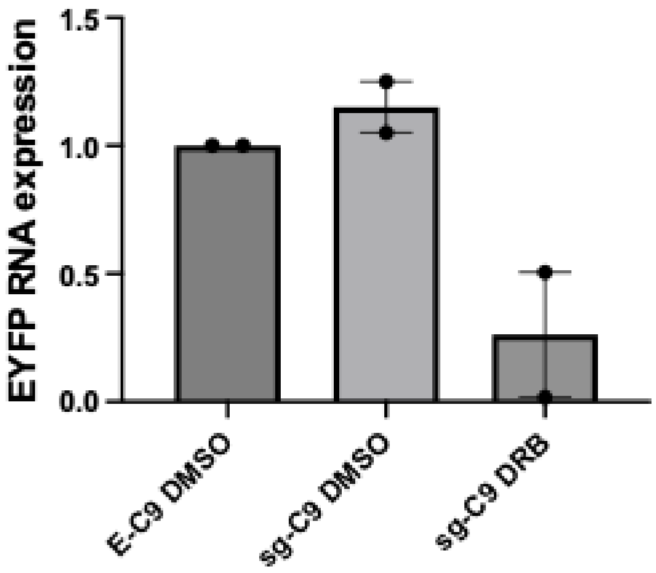

(4) Given that the number of GFP/YFP-positive cells is low, the results are likely sensitive to the gating strategy employed. The description of the gating strategy, including the controls used, should therefore be expanded in the Materials and Methods section.
We have expanded this part in the Materials and Methods section.

Reviewer #3 (Remarks to the Author):

This manuscript by di Lillo et al. investigates the impact of a site-specific DNA double-strand break (DSB) on RNA and protein expression. The authors establish two reporter systems: one in HeLa cells, in which a GFP construct lacking a canonical promoter is randomly integrated into the genome together with an upstream viral LTR, and a second in mouse embryonic fibroblasts (MEFs), where a promoterless EYFP reporter is inserted into the Rosa26 locus and transcription is blocked by a Lox-Stop-Lox cassette. In both systems, Cas9-induced DSBs upstream of the reporter lead to transcription and translation of GFP/EYFP in a small fraction of cells.

The authors conclude that DSB induction enables transcription of the GFP/EYFP constructs. These observations are very interesting and consistent with emerging evidence that DNA breaks can stimulate transcription in cis. Importantly, the authors go beyond previous work by proposing that the DSB itself can function as a promoter capable of driving gene expression and protein synthesis. The idea that transcription could initiate directly from the break site, without the need for a pre-existing promoter to assemble the transcriptional machinery, is conceptually exciting. While I find the conceptual framework compelling and the observations impactful, I believe that the conclusion that DSBs can act as a functional promoter is not yet fully supported by the experimental evidence presented. In particular, the potential contribution of upstream

promoter or regulatory elements to GFP/EYFP expression upon break induction has not been sufficiently explored. Below, I outline some experiments that would substantially strengthen the paper.

Major points:

1. As an internal control, it would be important to test whether inducing a Cas9-mediated DSB at an unrelated genomic locus leads to GFP/EYFP expression in the GΔP/EYFP reporter cells. This experiment would help establish the specificity of reporter activation to local break induction.
We agree with this reviewer and indeed this control is included in the manuscript and consists of the CCR5 condition used in both the experimental systems. In these settings, Cas9 carries a sgRNA targeting the C-C chemokine receptor 5 (CCR5) gene, whose biallelic inactivation does not impair cell survival and proliferation. Importantly, the DNA damage induced at this locus does not trigger GFP or YFP transcription (see Figures 1 and 2), supporting the specificity of reporter activation in response to local break induction.

The main conclusion of the manuscript is that transcription of the GFP/EYFP reporters is initiated directly at the DSB site, independently of upstream promoter activity. However, in both reporter systems, the break is introduced near known regulatory elements (a viral LTR in HeLa cells and the Rosa26 locus in MEFs), raising the concern that DSB induction could indirectly increase transcription from these upstream elements rather than initiating transcription de novo at the break site. While the authors show long-read sequencing of the HeLa construct after sg-Cas9 cutting, similar analyses are not presented for the Rosa26 locus. Several experimental controls could strengthen this claim:

2. The authors should test whether break induction affects transcription upstream of the cut site. RT–qPCR of regions upstream of the DSB (near the LTR in HeLa and the Rosa26 promoter in MEFs) after Cas9 cutting would determine whether basal transcription from these loci increases. Complementary analysis of RNA polymerase II occupancy across the integrated reporter region, including upstream sequences, the cut site, and the reporter gene, by Pol II ChIP would further clarify whether transcription initiates at the break or is enhanced from upstream promoters.
Analysis of RNA polymerase II occupancy across the integrated reporter region by Pol II ChIP is technically challenging and perhaps unfeasible because the locus is quite small and crowded with genetic elements. Therefore, ChIP signals should be highly resolved, in practice meaning that chromatin fragments should be smaller than 120 bp and such small and narrow fragmentation by sonication is hard to achieve in our experience. In the absence of such resolution, it would not be possible to precisely discriminate RNA polymerase II occupancy at the break site from occupancy at upstream promoter elements.
Rather, we performed a functional assay measuring transcription by ONT sequencing that we think is more informative.
Regarding whether break induction affects transcription of the LTR sites in the HeLa GΔP cell system, this information was already shown in Figure 1D (reported also here below), which indicates that transcription is not impacted by the generation of DNA damage (see the red box).

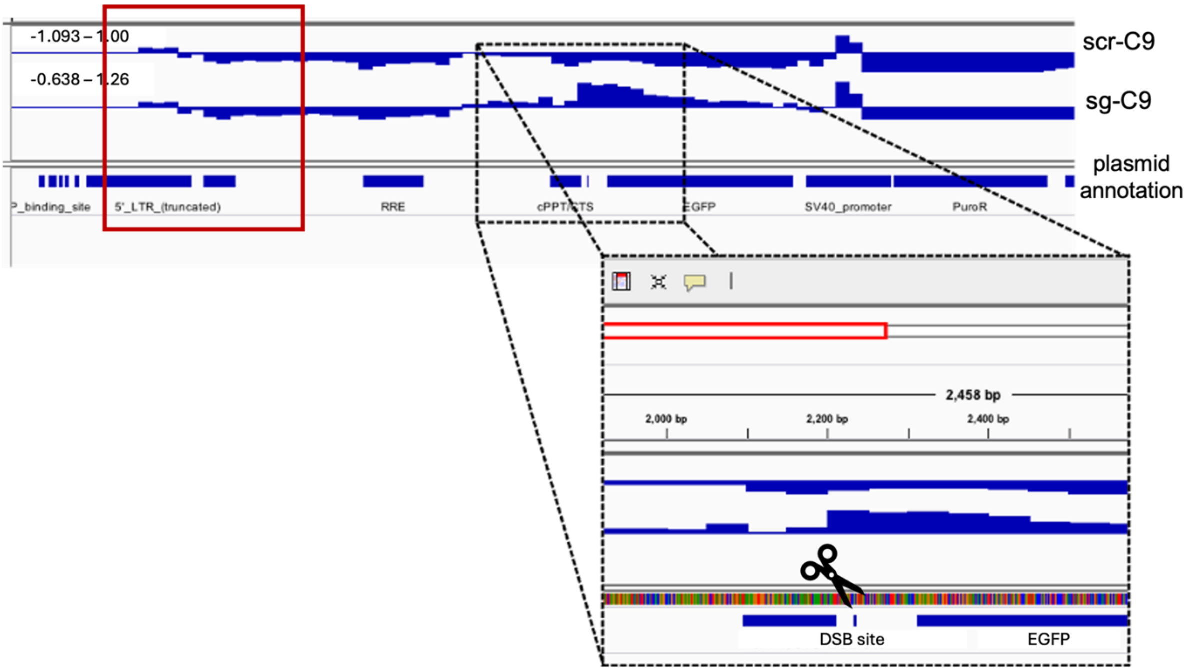

Since the MEF R26 EYFP cell system is a cleaner model, as also recommended by our reviewers, we have now performed a direct cDNA sequencing exploiting Oxford Nanopore Technology (ONT) in these cells in duplicate. We have analyzed three different time points (4, 8 and 12 days post infection) and we confirmed that, upon DSB generation upstream of the EYFP gene, EYFP transcripts starting from the polyA region were induced at all timepoints, with the control scr-C9 condition, that did not show any read at the EYFP locus, as a reference (now Figures 2E,F and S2E). In this setting, Cas9 delivery is associated with a slight decrease of reads of the NeoR/KanR gene (the gene immediately upstream the EYFP region) in the sg-C9 samples compared to the scr-C9 condition (Figure S2E, also reported here below), consistent with downregulation of pre-existing transcription upon DSB generation as we also previously reported in <u>Iannelli et al, Nat Comm 2017</u>.

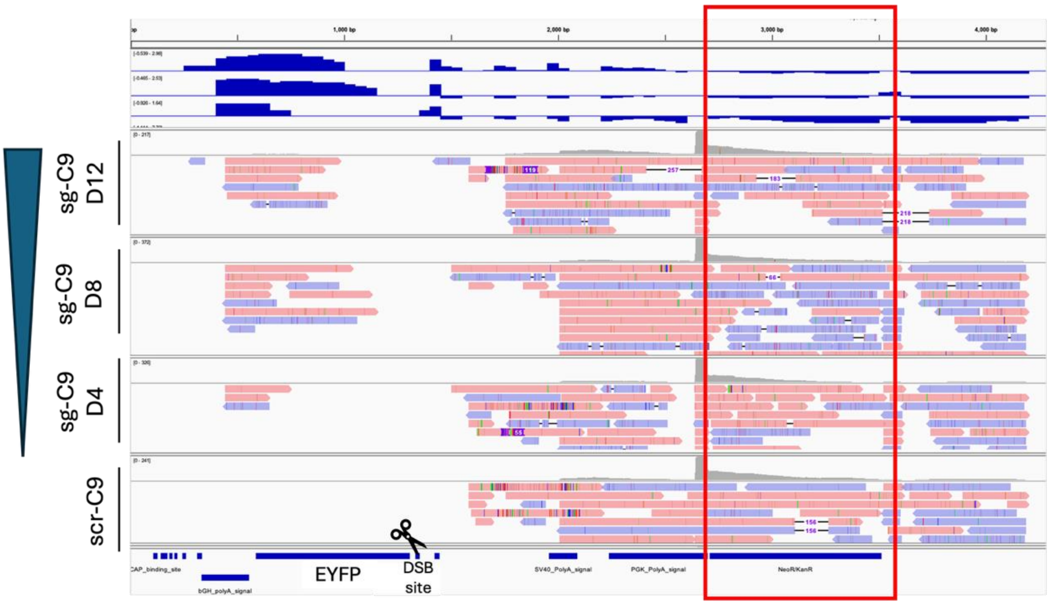

3. The authors use long-read RNA sequencing of the HeLa GΔP construct to distinguish transcripts originating from the upstream LTR versus those initiated at the DSB. However, similar analyses are not presented for the Rosa26 EYFP reporter. Performing comparable long-read RNA sequencing for the Rosa26 locus would allow the authors to determine whether transcription in this system also arises independently of the upstream promoter, thereby strengthening the central claim that break site acts as a functional promoter.
We have now performed this experiment, and the results have also been used to address point 2 of this referee.

4. In Figure 3, Cas9 is transiently expressed, yet EYFP RNA accumulation is detected several days later, when breaks are likely repaired. This contrasts with previous studies reporting rapid and transient transcriptional responses shortly after DSB induction. To better connect EYFP activation with the DNA damage response, the authors could test whether short-term treatment with Mirin, an MRE11 inhibitor known to suppress DSB-induced transcription, affects EYFP RNA levels.
In previous publications from our laboratory, we have treated cells with mirin (an inhibitor of MRE11), demonstrating that damage-induced transcription depends on the MRN complex (Michelini et al NCB 2017; Pessina et al NCB 2019; Sharma et al Cell Reports 2021). However, in those studies, the experimental setups used involved a temporally controlled and relatively short window of DNA damage generation, compatible with acute mirin treatment. In contrast, even the fastest experimental design described in this manuscript – based on the RNP-based approach – still requires sustained DNA cleavage for approximately 24 hours before damage-induced transcripts can be robustly detected. Nevertheless, we attempted to follow this referee's suggestion, and in line with our previous experimental settings, we treated cells with mirin (30 minutes) prior to RNP transfection and collected samples 24 hours later. Under these conditions, cells were therefore exposed to mirin for 24.5 hours. Such prolonged treatment resulted to be toxic, preventing subsequent analysis of the samples.

5. The authors state that high-depth sequencing of sg-Cas9 EYFP$^+$ samples did not reveal significant rearrangements or large-scale genomic alterations, but these data are not shown. Given the low frequency of EYFP-positive cells, it is important to more rigorously exclude the contribution of rare recombination or structural rearrangements at the reporter locus. Providing the sequencing data or complementary analyses (e.g. DNA-FISH with two differently labeled probes flanking the cut site) would strengthen the conclusion that reporter activation reflects local DSB-induced transcription rather than recombination-based events.
We agree with the referee that, given the relatively low frequency of EYFP-positive cells, it is relevant to exclude the possibility that reporter activation results from rare recombination events or structural rearrangements at the reporter locus rather than from local DSB-induced transcriptional activation. To address this, we performed ultra–high-depth sequencing of the EYFP_R26 locus (nucleotides 6604–9574), achieving a median coverage of depth 276,042× in the untreated sample and 269,578× in the sg-Cas9 EYFP$^+$ sample. Reads were aligned using BWA-MEM and per-base allele counts were extracted with samtools mpileup according to previously established analytical pipelines. This sequencing depth provides a conservative limit of detection of approximately

0.1-1% variant allele frequency. Within this sensitivity range, we did not detect recurrent or enriched sequence configurations indicative of rearrangements within the interrogated locus.
In addition, in an independent analysis, PCR amplification of the reporter region consistently yielded a single specific product without additional bands or smearing (Figure S4B), arguing against the presence of recombination events generating alternative amplicon structures. Although across the entire covered interval minor subclonal variants (fractional abundance below 6%) were observed confined to few short regions of the locus, in two of these regions both sg-Cas9 EYFP^{+} and untreated samples displayed identical genomic architecture and comparable allele frequencies, excluding structural divergence between conditions with any functional consequences (Figure 4).
These data indicate that there is no detectable molecular evidence supporting structural rearrangements at the reporter locus.
Overall, our results strongly support the conclusion that EYFP activation reflects local DSB-associated transcriptional effects rather than recombination-driven reporter reconstitution. For completeness and transparency, we have however now explicitly added to the revised manuscript a statement acknowledging that rare structural events occurring outside the amplified interval cannot be entirely excluded within the current experimental framework.

Minor points:

6. For clarity, it would be helpful to include the schematic representations of the reporter constructs and the positions of the qPCR primers in the main figures rather than only in the Supplementary Information. This would make it easier for readers to follow the experimental design and data interpretation.
We have revised the figure accordingly.

7. The authors report the copy number of the HeLa GΔP reporter (~18 copies per cell), but comparable information is not provided for the WT-GFP construct, which is used side-by-side with GΔP in subsequent experiments. Reporting WT-GFP copy number is important to exclude potential effects of unequal integration on basal or DSB-induced expression.
We have now added this information in the manuscript in the Figures 1B and 1F.

8. Related to the previous point, for the selected GΔP “clone 1” with low basal transcription, it would be important to indicate the reporter copy number, as this could influence both basal and DSB-induced transcription.
In the previous version of this manuscript, we estimated the number of EGFP copies in HeLa cells by qPCR, assuming that the actin gene is present in two copies per cell. However, thanks to the comments of referee 1, we now know that HeLa cells contain three copies of the actin gene. Given the high degree of aneuploidy of these cells, we decided not to indicate EGFP copy number but to show the results as the fold change. Based on this analysis, in the HeLa GΔP cl1, the EGFP gene is three folds more abundant than the actin gene.
Importantly, we believe that reporter copy number does not influence DSB-induced transcription as similar results were obtained in both HeLa GΔP cell system (based on multiple integrations of the reporter gene) and MEF R26 EYFP cell system (based on a single copy integration of the reporter gene).

9. The way the authors present the read coverage in Figure S1L is confusing. In Figure S1L, the scale of the coverage is not indicated, and the graph for the scr-Cas9 control appears truncated, making it difficult to compare conditions. In addition, the read coverage for the LTR region is not shown or indicated in the schematic. Including the LTR coverage and marking its position in the scheme, along with a clear y-axis scale for all traces, would improve clarity and help readers better interpret the data.
The coverage was indicated in the original figure, but at low resolution, we have now improved it. We thank the referee for pointing this out.
One of the two LTR regions is already reported in Figure 1D, while in Figure S1L we focus specifically on the region around the break that is the most relevant for interpreting damage-induced transcription. Here we show that reads were already present in the control condition before DNA damage generation, whereas after cutting the majority of reads initiate at or very close to the break. The quantitative analysis supporting this observation is reported in the main figure (Figure 1E).
